# Rice husk–based pyrogenic carbonaceous material efficiently promoted peroxymonosulfate activation toward the non-radical pathway for the degradation of pharmaceuticals in water

**DOI:** 10.1007/s11356-023-30785-1

**Published:** 2023-11-22

**Authors:** Marcela Paredes-Laverde, Jazmín Porras, Nancy Acelas, Jhonnaifer J. Romero-Hernández, Sindy D. Jojoa-Sierra, Lázaro Huerta, Efraím A. Serna-Galvis, Ricardo A. Torres-Palma

**Affiliations:** 1 Grupo de Investigación en Remediación Ambiental y Biocatálisis (GIRAB), Instituto de Química, Facultad de Ciencias Exactas y Naturales, Universidad de Antioquia UdeA, Calle 70 No. 52-21, Medellín, Colombia; 2 Grupo de Investigaciones Biomédicas Uniremington, Facultad de Ciencias de La Salud, Corporación Universitaria Remington (Uniremington), Calle 51 No. 51-27, Medellín, Colombia; 3 Grupo de Materiales Con Impacto, Facultad de Ciencias Básicas, Universidad de Medellín, MAT&MPAC, Medellín, Colombia; 4 Instituto de Investigaciones en Materiales, Universidad Nacional Autónoma de México, A.P. 70-360, 04510 Ciudad de México, México; 5 Grupo de Catalizadores y Adsorbentes (CATALAD), Instituto de Química, Facultad de Ciencias Exactas y Naturales, Universidad de Antioquia UdeA, Calle 70 # 52-21, Medellín, Colombia

**Keywords:** Analgesic degradation, Persulfate activation, Rice husk wastes, Singlet oxygen, Water treatment

## Abstract

Pristine pyrogenic carbonaceous material (BRH) obtained from rice husk and modified with FeCl_3_ (BRH-FeCl_3_) were prepared and explored as carbocatalysts for the activation of peroxymonosulfate (PMS) to degrade a model pharmaceutical (acetaminophen, ACE) in water. The BRH-FeCl_3_/PMS system removed the pharmaceutical faster than the BRH/PMS. This is explained because in BRH-FeCl_3_, compared to BRH, the modification (iron played a role as a structuring agent mainly) increased the average pore diameter and the presence of functional groups such as -COO^−^, -Si–O^−^, or oxygen vacancies, which allowed to remove the pollutant through an adsorption process and significant carbocatalytic degradation. BRH-FeCl_3_ was reusable during four cycles and had a higher efficiency for activating PMS than another inorganic peroxide (peroxydisulfate, PDS). The effects of BRH-FeCl_3_ and PMS concentrations were evaluated and optimized through an experimental design, maximizing the ACE degradation. In the optimized system, a non-radical pathway (i.e., the action of singlet oxygen, from the interaction of PMS with defects and/or -COO^−^/-Si–O^−^ moieties on the BRH-FeCl_3_) was found. The BRH-FeCl_3_/PMS system generated only one primary degradation product that was more susceptible to biodegradation and less active against living organisms than ACE. Also, the BRH-FeCl_3_/PMS system induced partial removals of chemical oxygen demand and dissolved organic carbon. Furthermore, the carbocatalytic system eliminated ACE in a wide pH range and in simulated urine, having a low-moderate electric energy consumption, indicating the feasibility of the carbocatalytic process to treat water polluted with pharmaceuticals.

**Supplementary Information:**

The online version contains supplementary material available at 10.1007/s11356-023-30785-1.

## Introduction

Pyrogenic carbonaceous material (PCM) can be produced by thermochemical conversion and has some organic carbon level content (Lehmann and Joseph 2015). PCMs are used as catalysts to produce reactive oxygen species (ROS) from the activation of inorganic peroxides for degrading organic pollutants, which has been recognized recently as an advanced oxidation process (AOP) (Huang et al. 2021a). This class of AOP (named carbocatalysis) has increased in popularity in the last years due to its accessibility, elevated recoverability, and simple synthesis method to obtain carbocatalytic materials, in addition to the high degradation efficiency (Scaria et al. 2022). In the carbocatalytic systems, the ROS are generated from the activation of oxidizing agents such as hydrogen peroxide (H_2_O_2_), peroxymonosulfate (PMS), or peroxydisulfate (PDS) by the PCMs.

Carbon nanotubes, carbon fibers, graphene, fullerene, activated carbon, and biochar are PCMs commonly employed in carbocatalysis (Hosseini et al. 2021). However, other materials with low carbon content do not have a specific name and keep their general classification of PCM (Hagemann et al. 2018). In general, the PCMs can be produced from agro-industrial wastes such as cotton stalks, sugarcane residues, and corn stems, which are widely used in water remediation (Minh et al. 2020; Scaria et al. 2022). PCMs are very interesting materials because of the easy access to raw materials, residue valorization, and reduction of disposal waste problems. Besides, disordered amorphous carbon structure and the presence of oxygenated functional groups allow to PCM perform the activation of peroxides (Gao et al. 2020). However, in some cases, pristine PCM is not enough efficient by itself and its catalytic capability should be improved by modifying it with metals, metal oxides, or salts (Faheem et al. 2020). Therefore, FeCl_3_, an environmentally friendly and abundant transition metal salt (Su et al. 2018), can be used to produce functionalized PCM having good catalytic properties for the activation of PMS, PDS, or H_2_O_2_ (Li et al. 2021).

On the other hand, rice husk is an abundant agro-industrial waste produced worldwide, ~ 150 million tons per year, which represents a risk to the environment (Chen et al. 2021a). As an alternative to face this concern, the rice husk can be used as a precursor for the preparation of PCMs with and without metal charge. PCM from rice husk has been used as an activator of PDS and PMS for the removal of organic pollutants in water such as phenols (Hussain et al. 2017; Liu et al. 2021; Zhang et al. 2022), esters (Dong et al. 2019), dyes (Li et al. 2020; Xie et al. 2021), and pharmaceuticals (Avramiotis et al. 2021a, b; Xie et al. 2021). Indeed, biochar from rice husk modified with FeCl_3_ has been tested for PMS activation and bisphenol A elimination through radical pathways where competing effects by the matrix components were observed (Gao et al. 2022). Thus, the utilization of rice husk–based PCM in search of rapid PMS activation toward selective routes, such as the non-radical ROS, should be explored. Moreover, the biodegradability and biological activity of the transformation products generated in the process and the target pollutant degradation in complex matrices must also be studied. Therefore, such lacking topics were developed herein. It is important to mention that a recent work reported the treatment of acetaminophen (ACE) by the activation of PMS with an iron-doped bone char through a non-radical pathway (Zeng et al. 2023). However, the composition, structure, and carbocatalytic behavior of that carbonaceous material are different from the obtained by using PCMs prepared from agro-industrial wastes of vegetal origin, as considered in our work.

This research presents the preparation of rice husk–based PCM functionalized with FeCl_3_ and its use as a catalyst to activate inorganic peroxides toward the formation of non-radical species. Initially, pristine PCM (BRH) and PCM modified with FeCl_3_ (BRH-FeCl_3_) were synthesized and compared as activators of PMS for the elimination of ACE in water. The results were explained through of physicochemical characteristics of the prepared PCMs. Then, the best carbocatalytic material was selected, the reuse cycles of this material were assessed, and the catalytic potential to activate PDS for the elimination of ACE was also established. Also, the BRH-FeCl_3_/PMS system was optimized. Thus, the operational parameters such as PCM dose and PMS concentration were evaluated through the response surface methodology. Afterward, the degradation mechanism of PMS activation toward the target pollutant was determined by the addition of specific scavengers, electron paramagnetic resonance (EPR), and XPS analyses. Moreover, a primary product generated in the carbocatalytic system was identified by using LC–MS. The biodegradability and biological activity of the product were determined by employing theoretical tools. Also, the phytotoxicity of the treated solution was measured. Finally, the efficiency of the BRH-FeCl_3_/PMS system to degrade ACE at a wide pH range (4–10) and a complex matrix (simulated fresh urine), plus the electric energy consumption (EEC), were evaluated.

## Material and methods

### Reagents

Acetaminophen (ACE, C_8_H_9_NO_2_) was supplied by Laproff (Medellín, Colombia). This compound was chosen as a model of pollution by pharmaceuticals since it is one of the most commonly used painkillers in the world and has been found in a variety of water sources (Zhao et al. 2018; Botero-Coy et al. 2018; Patel et al. 2019; Phong Vo et al. 2019), which cause negative impacts on environmental systems and human health (Fisher and Curry 2019; Phong Vo et al. 2019). Sodium peroxydisulfate (PDS, Na_2_S_2_O_8_) was purchased from Fisher Scientific (England, UK). Oxone, which is the source of potassium peroxymonosulfate (PMS, KHSO_5_), was distributed by Sigma-Aldrich (St Louis, USA). Other reagents such as hydrogen peroxide (H_2_O_2_) 30% (w/v), ferric trichloride (FeCl_3_), sodium hydroxide (NaOH), hydrochloric acid (HCl), formic acid (CH_2_O_2_), acetonitrile (C_2_H_3_N), methanol (CH_4_O), 4-benzoquinone (BQ), sodium azide (NaN_3_), urea (CH_4_N_2_O), potassium chloride (KCl), sodium dihydrogen phosphate (NaH_2_PO_4_), magnesium chloride (MgCl_2_), ammonium chloride (NH_4_Cl), calcium chloride (CaCl_2_), and sodium sulfate (Na_2_SO_4_) were acquired from Merck S.A. (Germany). Milli-Q water was utilized to prepare the HPLC mobile phase, and distilled water was used to make all other solutions.

### PCM preparation

Rice husk waste (RH) was collected from a local agroindustry in Huila (Colombia). Subsequently, the rice husk waste was pyrolyzed in a horizontal tube furnace (Resistencias y Equipos, Ref. HC, 3.6 kW, 220 V) in the presence of N_2_ gas (99.995%) using a flow rate of 100 mL per min. To carry out the pyrolysis process, the temperature was raised by 5 °C every minute to 500 °C and then maintained there for 2 h. Afterward, the pyrolyzed rice husk was modified with FeCl_3_, using a mass ratio metal salt/char of 3:1. This mixture was then heated up to 800 °C at a rate of 5 °C per min and then remained at 800 °C for an hour. After the modification, the final PCM was washed with distilled water until it reached pH neutrality, and it was dried for 24 h at 105 °C. Subsequently, the final solid product was macerated, sieved with a mesh 40 (420 µm), and stored and named BRH-FeCl_3_. For comparison purposes, another PCM (designated as BRH) was obtained in the same way but with no activation using FeCl_3_.

### Characterization techniques

BRH and BRH-FeCl_3_ were characterized using different techniques. Ashes and fixed carbon were determined by thermogravimetric analyses (TGA) in a Q500 TA Instruments equipment, using the described methodology in Paredes-Laverde et al. (2019). A CHSN/O elemental analyzer (Leco Truspec micro) was used for analyzing the content of O and C according to the standard method ASTM D-5373–08. The functional groups on these materials were identified using Fourier transform infrared spectroscopy (FTIR, from 4000 to 450 cm^−1^) by attenuated total reflectance (ATR), in a PerkinElmer-Waltham equipment. The solids addition method allowed us to get the point of zero charges (PZC) of BRH and BRH-FeCl_3_ (Guechi and Hamdaoui 2015). The textural properties of the PCMs were determined from nitrogen adsorption at − 196 °C, using Micrometrics ASAP 2020. The Brunauer–Emmett–Teller (BET) equation was used to calculate the specific surface area (*S*_BET_), and the average pore diameter (*D*_AP_) was obtained through the 4*V*_TP_/*S*_BET_ equation. The structure of the PCMs was determined by X-ray diffraction (XRD) in a Rigaku SmartLab X-ray diffractometer with Cu Kα1 radiation (*λ* = 1.5406 Ǻ), in the 2*θ* angle (range of 10 to 70°) with a step size of 0.02° and period of 50 s. Also, the crystallographic composition of materials was analyzed by a Rietveld refinement in Fullprot (free software), and the identification of carbonaceous material (CM), Fe_3_O_4_, and α-Fe was made using the ICSD collection code 76767, 158745, and 64999, respectively. Furthermore, to observe the morphology and identify the relative content of elements on the surface of the PCM samples, a scanning electron microscope coupled with an energy-dispersive spectrometer (SEM/EDS) of reference JEOL JSM-6490LV was used. Moreover, for the most efficient PCM in the carbocatalytic system, it was performed X-ray photoelectron microscopy (XPS) in a PHI 5000 VersaProbe II Scanning XPS microprobe using al-Kα X-ray as a source. The details of the XPS analyses are provided in Text SM 1  in the Supplementary Material (SM).

### Degradation experiments

Initially, the catalytic activity of BRH and BRH-FeCl_3_ was evaluated. These studies took place in a batch reactor, using 100 mL of ACE solution at 2.4 mg L^−1^, 0.2 g L^−1^ of PCM, and 0.5 mmol L^−1^ (mM) of PMS at natural pH (6.8), under constant stirring in a Dragon Lab magnetic stirrer MS-M-S10 model (power 0.02 kW) for 90 min. Control experiments were also carried out, under the same conditions, but without PCM or PMS. The PCM/PMS system with the most efficient elimination of the pollutant was selected. Subsequently, the ability to activate another inorganic peroxide (i.e., PDS) was tested. Also, the reuse of the carbonaceous material was evaluated by washing it with distilled water and drying it at 100 °C for four consecutive cycles.

The catalyst dose and PMS concentration were optimized through the response surface methodology using the Statgraphics Centurion XVII program (Table SM1, in the Supporting Material). The role of different ROS, in the ACE degradation, was examined using the PCM/PMS best system; experiments were performed using a pharmaceutical concentration of 2.4 mg L^−1^ (0.016 mM), with 1.6 mM of scavengers, 0.65 g L^−1^ of PCM, and 0.54 mM of PMS, at a reaction time of 3 min. Scavengers such as methanol were applied to quench ${\mathrm{HO}}^{\bullet }$ and ${\mathrm{SO}}_{4}^{\bullet -}$. To investigate the involvement of ${\mathrm{O}}_{2}^{\bullet -}$, ${{}_{}{}^{1}\mathrm{O}}_{2}$, BQ, and NaN_3_ were used, respectively. Also, to remove the dissolved oxygen, an additional experiment, bubbling the reaction system with N_2_ at 480 mL min^−1^, was done. Besides, the best PCM/PMS combination was evaluated in the degradation of ACE in a wide pH range (4.0, 6.8, 8.0, and 10.0) and the elimination of the target pharmaceutical in a simulated urine matrix. The simulated urine was prepared according to the recipe reported in Table SM2, based on a previous paper of our research team (Serna-Galvis et al. 2021).

### Analytical techniques

The ACE concentration was monitored with a UHPLC Thermo Scientific Dionex UltiMate 3000 instrument, equipped with a diode array detector at 243 nm and an Acclaim™ 120 RP-C18 column (5 μm, 4.6 × 150 mm). A 15:85 (v/v) acetonitrile/formic acid combination with a flow rate of 0.45 mL min^−1^ as the mobile phase. A volume of 20 μL was used for the injection. The transformation product was identified by LC–MS analysis in an LCMS TQ8060 instrument coupled to a triple quadrupole. Chromatographic conditions were similar to those used in the UHPLC equipment, and a scan range of 40–200 m/*z* was employed with the detector in positive ESI mode. In a Shimadzu TOC analyzer, the measurement of the concentration of dissolved organic carbon (DOC) was conducted. To determine the oxidizable organic matter, the chemical oxygen demand (COD) was carried out in an ECO 25-VELP thermo-reactor by the closed reflux method using potassium dichromate. The leaching of Fe from BRH-FeCl_3_ in the carbocatalytic process was monitored using a flame atomic absorption spectrophotometer (MK2 AA System-model M5) equipped with a graphite furnace and hydride generator.

Considering the possibility of future reuse of the treated water for crop irrigation, the phytotoxicity was evaluated by determining the Germination Index (G.I.) of mung bean (a commercial seed was used). For this purpose, ACE treated by BRH-FeCl_3_/PMS for 0, 3 (t), 6 (2t), and 9 min (3t) was considered. Then, 3 mL of sample from each experimental time was added to a petri dish containing four mung bean seeds. Distilled water was used as a positive control of germination, while glyphosate was a negative control of germination. After 5 days, the length of each germinated was measured, and the method proposed by Hoekstra et al. (2002) for the calculation of G.I. was utilized. In addition to the experimental determination of phytotoxicity, some environmental characteristics of the identified degradation by-product were assessed employing theoretical tools. Then, for such a by-product, the theoretical biotransformation pathways were obtained using the EAWAG/BBD prediction system (EAWAG 2022), and to analyze the potential biological activity, the PASS software program (free online version (W2D Team—PharmaExpert 2021)) was used.

To confirm the ROS formation in the BRH-FeCl_3_/PMS system, an analysis of electron paramagnetic resonance (EPR) was performed using a Bruker E580 spectrometer operating at X-band microwave frequency (9.8 GHz). The ${\mathrm{HO}}^{\bullet}$ and ${\mathrm{SO}}_{4}^{\bullet -}$ were trapped by 5,5-dimethyl-1-pyrroline N-oxide (DMPO), ${\mathrm{O}}_{2}^{\bullet -}$ was trapped by 5-(diethoxyphosphoryl)-5-methyl-1-pyrroline-N-oxide (DEPMPO), and ${{}_{}{}^{1}\mathrm{O}}_{2}$ was trapped by 2,2,6,6-tetramethyl-piperidinyloxyl (TEMP). The EPR device was run at room temperature, under non-saturating conditions of microwave power (10 mW) and 0.1 mT modulating field.

### Electric energy consumption

The electric energy consumption (EEC), for the BRH-FeCl_3_/PMS system utilized in the ACE elimination in simulated urine matrix and distilled water, was calculated. The EEC is the electrical energy required to achieve a determined pollutant removal (Torres et al. 2007), which can be calculated using the following equation:

1 $$EEC= {~}^{\left[P \times t \times 1000\right] }\!\left/ \!{~}_{\left[V \times 60 \times \mathrm{log}\left({C}_{i}/{C}_{f}\right)\right]}\right.$$

where ${C}_{i}$ and ${C}_{f}$ are the initial and final concentrations of ACE, respectively, *P* is the electric power (kW) used in the system (i.e., the electric power consumed by the stirrer in the case of carbocatalysis), and *V* is the volume (L) of water treated in time *t* (min).

## Results and discussion

### Evaluation of the ability of PCMs to activate PMS for the degradation of ACE

**Fig. 1:**
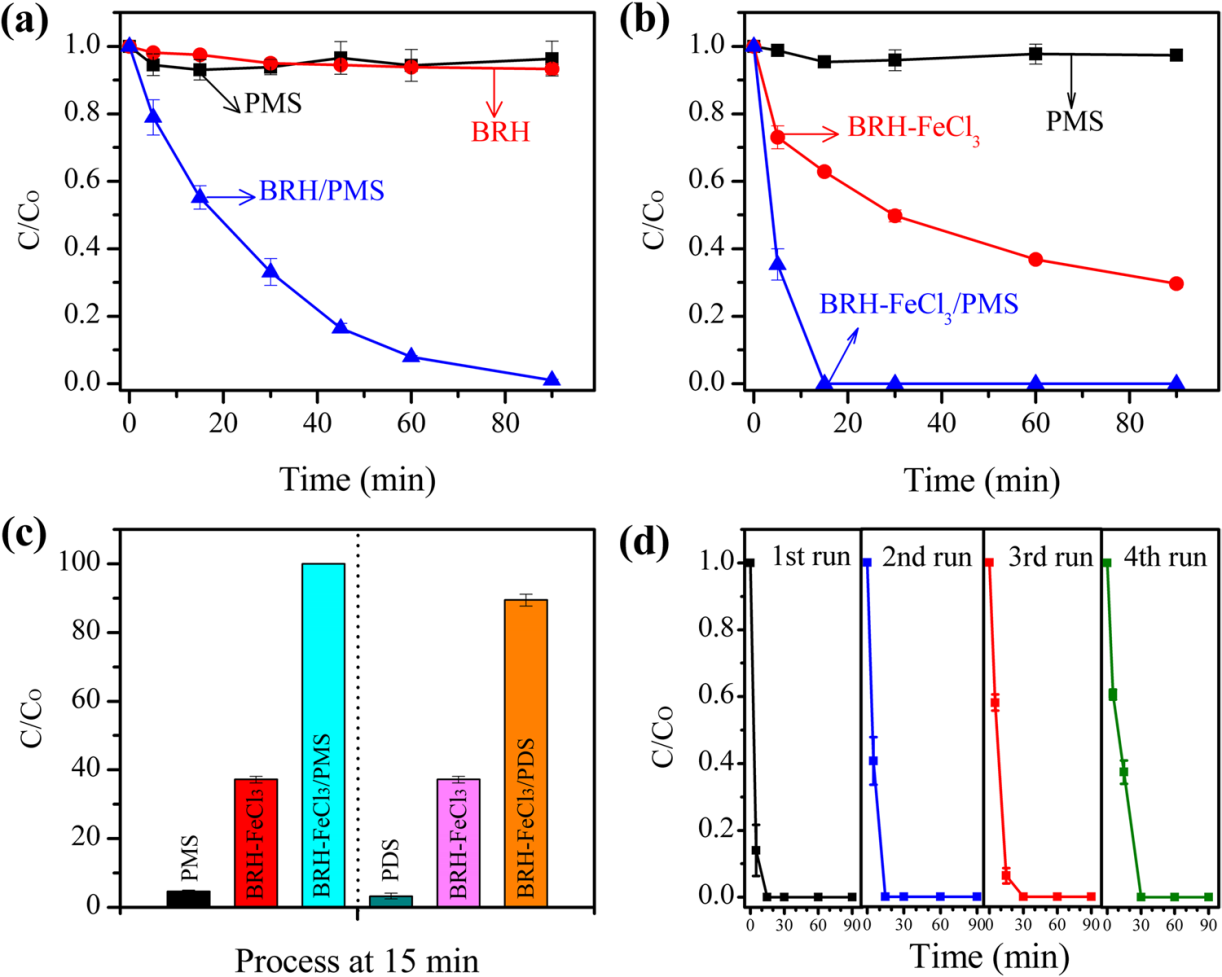
Evaluation of the catalytic properties of PCMs. **a** ACE elimination using BRH with PMS; **b** ACE elimination using BRH-FeCl_3_ with PMS; **c** ACE elimination using BRH-FeCl_3_ with PMS and PDS; **d** reusability of the BRH-FeCl_3_ in the ACE elimination using PMS as the oxidizing agent. Conditions: [ACE] = 2.4 mg L^−1^, [PCM] = 0.2 g L^−1^, [Oxidizing agent] = 0.5 mM, pH = 6.8

BRH and BRH-FeCl_3_ were initially evaluated for the activation of PMS to degrade ACE in water (Fig. 1). Also, the two carbocatalytic systems (i.e., BRH/PMS and BRH-FeCl_3_/PMS) were compared with the corresponding control subsystems (i.e., adsorption on the PCMs and direct action of PMS alone). The direct degradation of ACE by PMS (without PCM) showed an elimination lower than 10% (Fig. 1a), indicating that under the working conditions, the oxidizing agent alone had low degrading action on the target pollutant.

**Fig. 2:**
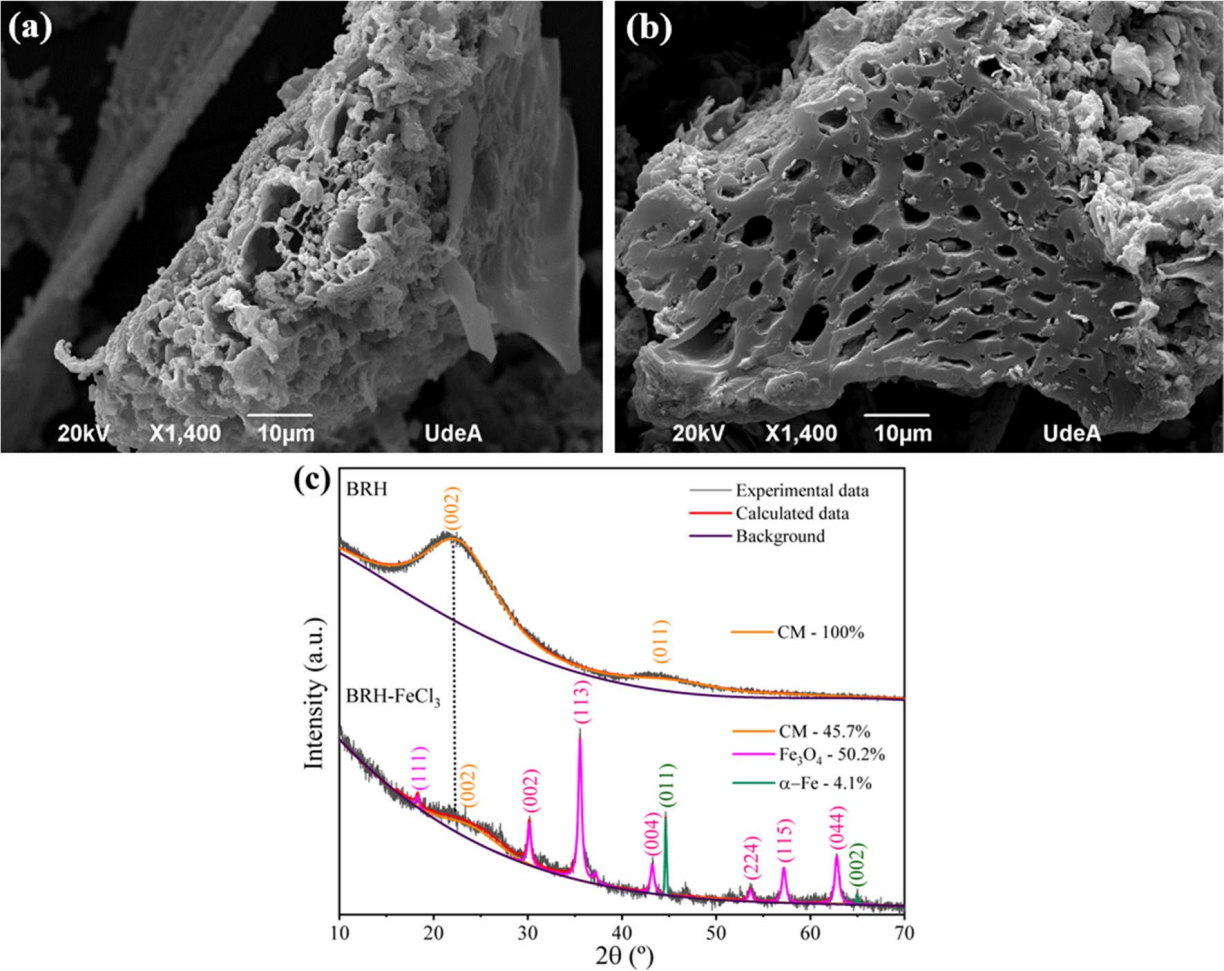
Morphological and structural analyses of the PCMs. **a** SEM micrography of BRH; **b** SEM micrography of BRH-FeCl_3_; and **c** XRD analysis for both PCMs

The adsorption controls indicated that BRH did not adsorb ACE (Fig. 1a), whereas BRH-FeCl_3_ removed ~ 70% of the pollutant in 90 min (Fig. 1b). These results are explained considering the physicochemical characteristics of the two carbonaceous materials (Table 1, the low content of elemental carbon and consequently a poor fixed carbon in BRH and BRH-FeCl_3_ indicate that these materials should appropriately be named PCMs). In addition to the C content in the carbonaceous materials, other characteristics must be considered in the adsorption process. Although BRH presented the highest *S*_BET_, BRH-FeCl_3_ developed a superior *D*_AP_, which allowed ACE to easily enter the pores of this last material. These results are also supported by the SEM analyses, which showed a rough and poorly porous surface for BRH (Fig. 2a), while BRH-FeCl_3_ stood out for its abundant porosity (Fig. 2b) useful for adsorbing ACE.

**Table 1:** Physicochemical characteristics of the PCMs

PCM	PZC		N_2_ physisorption		Elemental analysis (wt%)			Proximate analysis—dry base (wt%)			EDS analysis (wt%)		
			*S*_BET_ (m^2^ g^−1^)	*D*_AP_ (nm)	C	O		Fixed carbon	Ashes		Si	Fe	Cl
BRH	8.10		342	3.08	28.1	18.9		28.9	56.9		21.5	0.650	-
BRH-FeCl_3_	5.80		223	3.41	27.3	36.5		1.40	85.2		12.3	31.8	0.540
FTIR analysis													
Wavenumber (cm^−1^)		Functional group					BRH			BRH-FeCl_3_			
3100		-OH (Carboxyl and phenol)					Present			Increased			
2924		C-H (sp^3^-aliphatic carbon)					Present			Increased			
2147		C = N					Present			Similar			
1830		C = O					Present			Similar			
1630		C = C (sp^2^-aromatic rings)					Present			Increased			
1010		Si–O-Si					Present			Similar			
800		C-H (aromatic stretch)					Present			Similar			
580		Fe–O					Absent			Present

As the PZC value for both materials (Fig. SM1 and Table 1) was lower than the pKa for ACE (pKa 9.4 (Patel et al. 2021)) and at the pH of the experiment ACE is neutral, the adsorption by electrostatic charges attraction between the pollutant and adsorbent did not occur. However, according to the FTIR analysis reported in Table 1, the modification of BRH to produce BRH-FeCl_3_ increased the presence of functional groups such as C=C (aromatics) and -OH (phenol and carboxyl moieties), which may form π-π interactions and hydrogen bonds with ACE, respectively; thus favoring the adsorption process on BRH-FeCl_3_ (Paredes-Laverde et al. 2019).

The treatment of the model pharmaceutical by the BRH/PMS system showed a complete ACE removal at 90 min, while the BRH-FeCl_3_/PMS system removed all the pollutant after only 15 min of treatment. These results show that BRH-FeCl_3_/PMS has superior action on the pharmaceutical due to that, in addition to higher adsorption, the BRH-FeCl_3_ material can activate PMS toward species that promote the degradation of the target pollutant.

In fact, the comparison of BRH-FeCl_3_ and BRH indicates that the former has higher O content that is linked to the increase in oxygenated functional groups such as carboxyl and phenols (according to the FTIR results in Table 1), which could play a relevant role in the PMS activation. Furthermore, the modification of the carbonaceous material with FeCl_3_ increased groups C=C (sp^2^- aromatic rings) and C-H (sp^3^-aliphatic carbon) plus a high content of ashes according to proximate analysis (dry base; Table 1), which is also consistent with the presence of elements such as Fe (as revealed by the XRD analysis in Fig. 2c). According to the XRD results, the iron in BRH-FeCl_3_ can be found mainly as Fe_3_O_4_ and a smaller amount of the form α-Fe. It can be remarked that after modification, a significant percentage of carbonaceous content (45.7%) was preserved. The presence of iron and other species such as Cl and Si is also associated with the abundant ashes, which was also corroborated by the EDS and FTIR analyses (Table 1). Therefore, from this series of changes caused by the modification, it is possible to suggest that the -COOH, -OH (Li et al. 2022), C (sp^2^ and sp^3^), Fe (Kohantorabi et al. 2021), Si (Duan et al. 2020), and Cl (Gao et al. 2022) present in the BRH-FeCl_3_ material could participate in the PMS activation to produce ROS responsible for the fast ACE degradation observed in Fig. 1b.

Considering the best performance of BRH-FeCl_3_, this carbonaceous material was selected to develop the subsequent experiments in this study. Therefore, the ability of BRH-FeCl_3_ to activate another peroxide (PDS) was tested and compared to the system involving PMS. Figure 1 c shows that the BRH-FeCl_3_/PMS system eliminated the pollutant faster than the combination of BRH-FeCl_3_ with PDS (BRH-FeCl_3_/PDS). At 15 min of treatment, the BRH-FeCl_3_/PMS system showed a better synergy than BRH-FeCl_3_/PDS (Fig. SM2). The higher efficiency for the ACE elimination by the BRH-FeCl_3_/PMS system could be associated with the asymmetric structure of PMS compared to PDS (which is symmetric). Due to the asymmetry, there is a partial positive charge on the peroxide bond of PMS; then, it could be more susceptible than PDS to the activation by the BRH-FeCl_3_ toward ROS useful for the degradation of organic pollutants (Eqs. 2 and 3) (Grisales-Cifuentes et al. 2022).

2 $${\mathrm{BRH}-\mathrm{FeCl}}_{3}+\mathrm{PMS }\to \mathrm{ROS}$$

3 $$\mathrm{ROS}+\mathrm{ACE }\to \mathrm{Degradation\;products}$$

The reuse of the BRH-FeCl_3_ in the carbocatalytic system for the ACE degradation was also assessed. Therefore, the BRH-FeCl_3_ reuse was evaluated during four cycles (Fig. 1d). In the first and second reuse cycles of BRH-FeCl_3,_ 100% of the pollutant was eliminated in 15 min. In the third and fourth cycles, the process only required 30 min to remove 100% of ACE. The good results for BRH-FeCl_3_, even after four cycles of reuse, can be associated with the elevated catalytic capability of this material. This material seems to retain several active sites available to promote the continuous activation of PMS to degrade ACE (Nguyen et al. 2021).

### Effect of parameters (PMS concentration and BRH-FeCl_3_ dose) and evaluation of oxidation degree and mineralization extent during the treatment of ACE

The above results evidenced high efficiency and good reusability of the BRH-FeCl_3_ toward the PMS activation for the target pollutant degradation even under non-optimized conditions. Then, better results could still be expected under optimized conditions. Therefore, to optimize the degrading capability of the BRH-FeCl_3_/PMS system, an experimental design was applied (Grisales-Cifuentes et al. 2022). The BRH-FeCl_3_ dose and PMS concentration are key parameters to be evaluated in detail to maximize the ACE degradation. To achieve this, the effects dose of BRH-FeCl_3_ and the concentration of PMS were evaluated through the response surface methodology, using an experimental design (ED, as shown in Table SM1).

**Fig. 3:**
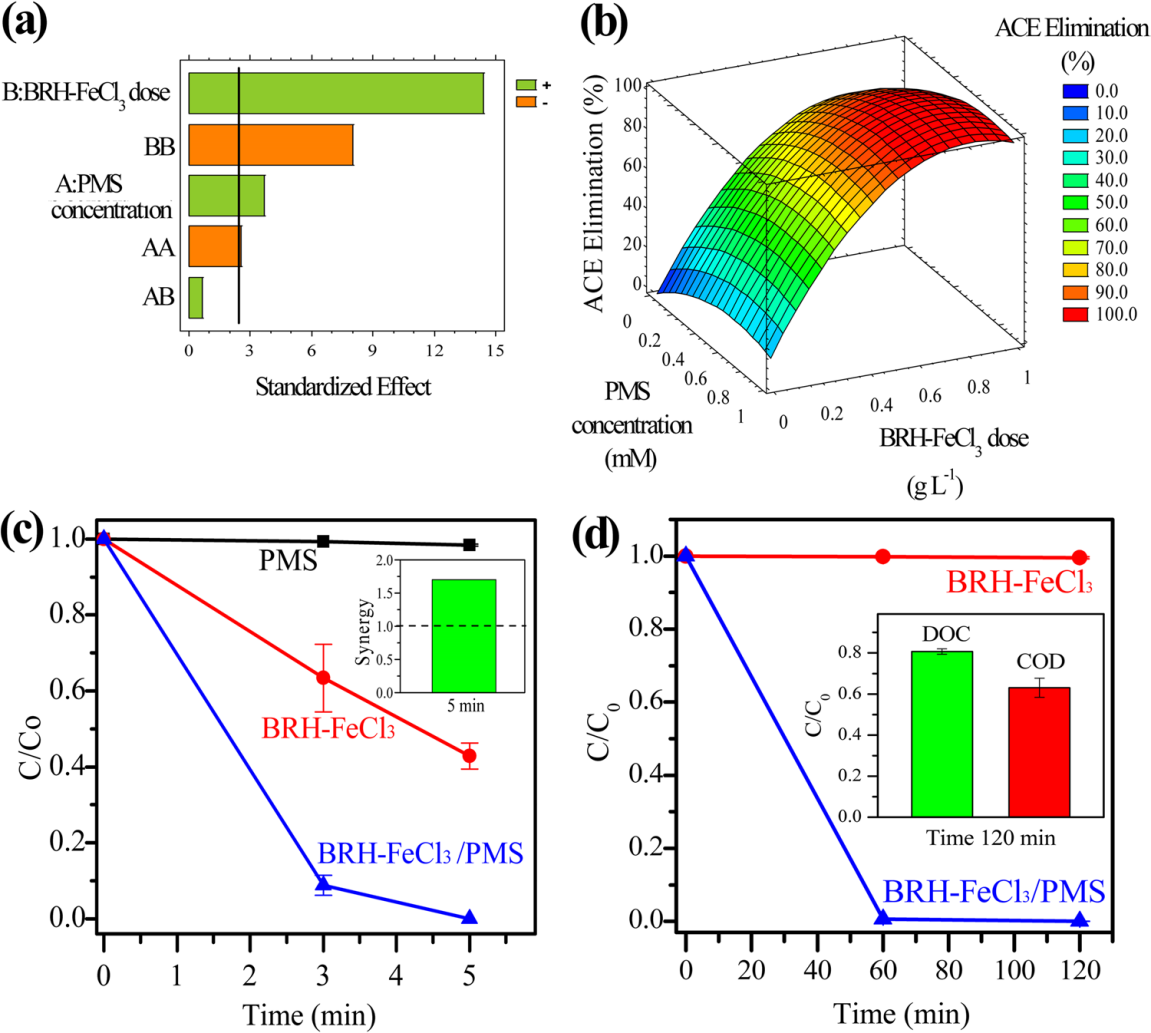
Evaluation of PMS concentration and BRH-FeCl_3_ dose in the ACE elimination. **a** Standardized Pareto diagram; **b** response surface diagram; **c** ACE degradation under optimal conditions derived from the experimental design (inset: synergy of the process, using ACE at 2.4 mg L^−1^, [BRH-FeCl_3_] = 0.65 g L^−1^, [PMS] = 0.54 mM, pH = 6.8); **d** DOC and COD analysis of process using an ACE concentration of 24 mg L^−1^ with [BRH-FeCl_3_] = 0.65 g L^−1^, [PMS] = 0.54 mM, pH = 6.8

Figure 3 a presents the Pareto diagram obtained from the ED, and this shows that the ACE elimination is effectively influenced by both tested variables and the interactions of variables that surpassed the vertical solid line of black color.

In detail, the BRH-FeCl_3_ dose (B) and PMS concentration (A), as well as the interaction between the two variables, have a positive effect on the response factor, demonstrating that ACE elimination improves when the respective variables are present in high amounts within the examined range. Meanwhile, the quadratic value of the BRH-FeCl_3_ dose (BB, which represents the effect of the interaction of the carbonaceous material with itself in the process) shows a negative effect in the ACE elimination. The quadratic value of PMS concentration (AA) also exhibited a negative impact, but it was not significant. Besides the Pareto diagram, the response surface was established (Fig. 3b), showing that if the quantity of BRH-FeCl_3_ is increased, the elimination of ACE improves. A similar trend was found for the PMS concentration in the process. However, when the concentration of PMS and BRH-FeCl_3_ doses are so high (i.e., the AA and BB interactions in Fig. 3a), the ACE degradation is decreased. A greater amount of BRH-FeCl_3_ provides more active surface sites for the production of ROS; consequently, the elimination of the pollutant is increased (Yin et al. 2019). If the amount of PMS increases, the elimination of ACE also increases due to a larger production of ROS (He et al. 2021). However, at very high BRH-FeCl_3_ doses, i.e., 0.80 g L^−1^, or very high concentrations of PMS (e.g., 1.00 mM), the elimination of the pharmaceutical is decreased due to the excess of carbonaceous material or PMS may interact with the produced ROS, scavenging them and causing detrimental/competence effects (Miao et al. 2021).

From the experimental design, it was also found that the optimal conditions to eliminate ACE require a BRH-FeCl_3_ dose of 0.65 g L^−1^ and a PMS concentration of 0.54 mM. The optimization was confirmed experimentally, and the corresponding results are reported in Fig. 3c. This figure shows that more than 90% of the pharmaceutical was removed after 3 min and all the pharmaceutical was eliminated in 5 min of treatment (presenting a synergy index of 1.7). These results demonstrated that the BRH-FeCl_3_ dose and PMS concentration can be properly controlled, thus improving the performance of the system, and avoiding additional operational expenses associated with high reagents consumption. Besides optimization, from the experimental design methodology, it was obtained a mathematical model (Eq. 4) that directly relates ACE elimination with the main influential variables (i.e., PMS concentration and BRH-FeCl_3_ dose). To verify the validity of Eq. 4, comparisons between the calculated and experimental values for the ACE elimination were made (Table SM1). It can be observed that the estimated values fit well with the experimental data. Indeed, the *R*^2^ value obtained from the software was 98%, confirming the good representation of the ACE degradation by the model equation.

4 $$\mathrm{ACE\;Elimination\;}\left(\%\right)=-40.5456\times {\left[\mathrm{PMS\;Concentration}\right]}^{2}-133.442\times {\left[\mathrm{BRH}-{\mathrm{FeCl}}_{3}\;\mathrm{Dose}\right]}^{2}+60.6286\times \left[\mathrm{PMS\;Concentration}\right]+215.641\times \left[\left[\mathrm{BRH}-{\mathrm{FeCl}}_{3}\;\mathrm{Dose}\right]-4.91014\right]$$

On the other hand, the oxidation and mineralization of pollutants are also important indicators of the performance of the degradation processes (Minh et al. 2020). Hence, the COD and DOC removals during the ACE degradation using BRH-FeCl_3_/PMS were appraised. A new set of experiments was developed by using a higher initial concentration of ACE (i.e., 24 mg L^−1^, because this amount allowed us to carry out the analytical measurements of COD and DOC). As seen in Fig. 3d, after 1 h of treatment, ACE was completely removed. To determine both oxidation and mineralization, the treatment was prolonged till 2 h. After such a time of treatment, initial COD and DOC decreased by 37% and 19%, respectively. These results indicate that the BRH-FeCl_3_/PMS system transformed the ACE into more oxidized products (COD was diminished), which can also be partially converted into water, carbon dioxide, and inorganic ions (as supported by the DOC decreasing). All these results confirm the carbocatalytic nature of the combination of BRH-FeCl_3_ with PMS.

### Identification of reactive species and active sites responsible for ACE degradation with BRH-FeCl_3_/PMS

**Fig. 4:**
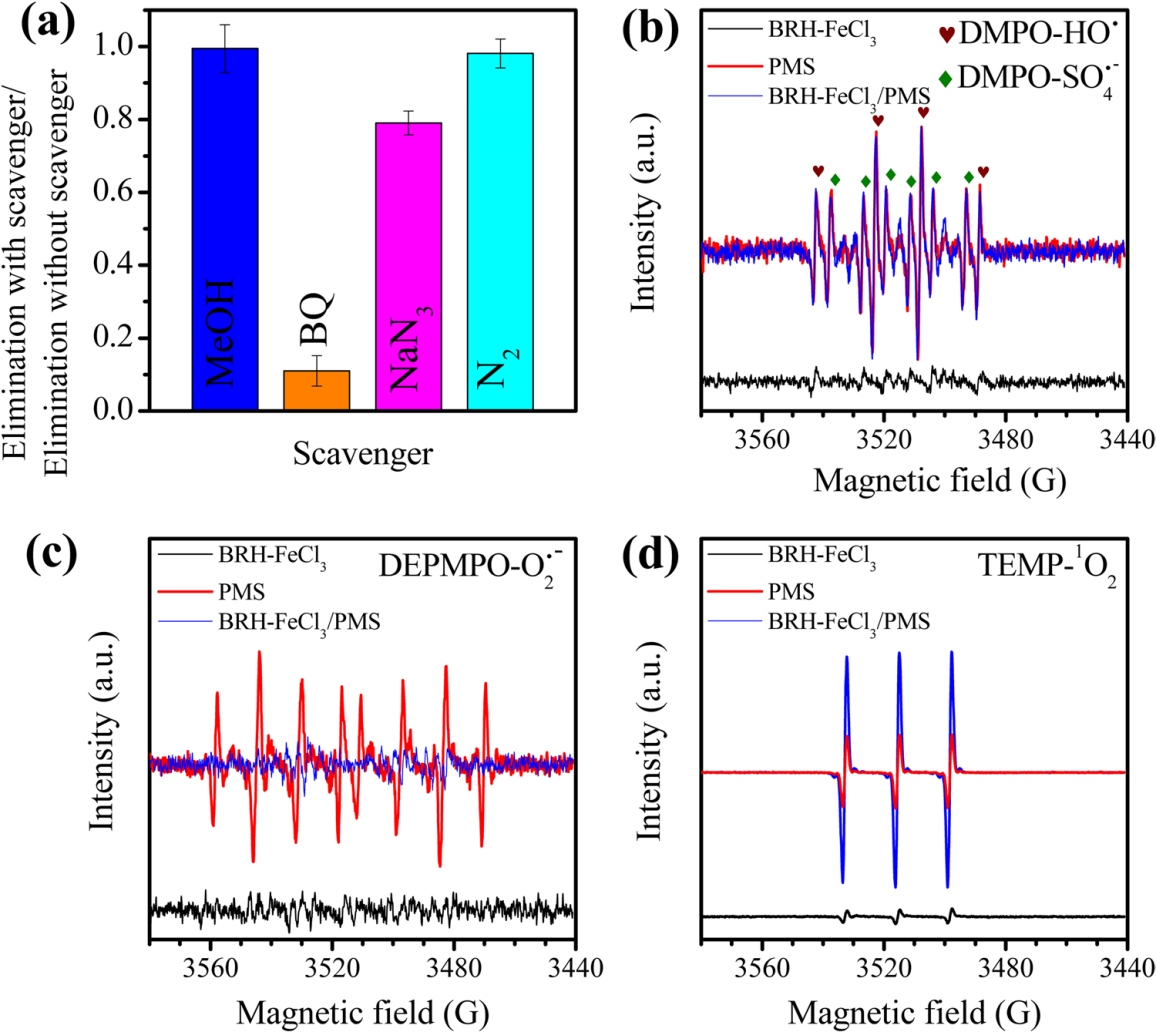
Reactive species involved in the ACE elimination by BRH-FeCl_3_/PMS. **a** Catalytic performance for ACE degradation at 2.4 mg L^−1^ (0.016 mM) under different scavenger presence (MeOH, BQ, NaN_3_ = 1.6 mM, N_2_ = 200 mL min^−1^) using [PMS] = 0.54 mM and [BRH-FeCl_3_] = 0.65 g L^−1^, time 3 min. Identification of ROS using EPR analysis: **b** DMPO: ${\mathrm{HO}}^{\bullet }$ and ${\mathrm{SO}}_{4}^{\bullet -}$; **c** DEPMPO: ${\mathrm{O}}_{2}^{\bullet -}$; **d** TEMP: ${{}_{}{}^{1}\mathrm{O}}_{2}$

In the carbocatalytic processes, ${\mathrm{SO}}_{4}^{\bullet -}$, ${\mathrm{HO}}^{\bullet }$, ${\mathrm{O}}_{2}^{\bullet -}$, and ${{}_{}{}^{1}\mathrm{O}}_{2}$ can be formed (Li et al. 2021). Therefore, to determine the participation of these species in our BRH-FeCl_3_/PMS system, experiments using scavengers were carried out. Methanol (MeOH), benzoquinone (BQ), and sodium azide (NaN_3_) were utilized as scavengers (at 100-fold the concentration of the pollutant; Fig. 4a).

It is reported that MeOH has a fast reaction toward both ${\mathrm{SO}}_{4}^{\bullet -}$ (${\mathrm{k}}_{{\mathrm{SO}}_{4}^{\bullet -} /\mathrm{MeOH}}$= 1.1 × 10^7^ M^−1^ s^−1^) and ${\mathrm{HO}}^{\bullet }$ (${\mathrm{k}}_{{\mathrm{HO}}^{\bullet }/\mathrm{MeOH}}$= 9.7 × 10^9^ M^−1^ s^−1^) (Zhang et al. 2021). Figure 4 a shows that there was no inhibition of ACE degradation in the presence of MeOH, indicating that both ${\mathrm{SO}}_{4}^{\bullet -}$ and ${\mathrm{HO}}^{\bullet }$ in solution were not relevant in the degradation of the pharmaceutical, and it also suggests the contribution of other reactive species in the process. Then, BQ was used to determine the involvement of ${\mathrm{O}}_{2}^{\bullet -}$ (${\mathrm{k}}_{{\mathrm{O}}_{2}^{\bullet -}/\mathrm{BQ}}$= 1.0 × 10^9^ M^−1^ s^−1^) (Ren et al. 2021) and NaN_3_ was used to detect the ${{}_{}{}^{1}\mathrm{O}}_{2}$ participation (${{\mathrm{k}}_{^{1}}}_{{\mathrm{O}}_{2}/\mathrm{NaN}_{3}}$= 2.5 × 10^9^ M^−1^ s^−1^) (Zhang et al. 2021). Figure 4 a shows a strong inhibition of ACE degradation by BQ but such an inhibition is caused by the adsorption of BQ on the BRH-FeCl_3_ surface (as demonstrated in Fig. SM3).

However, when NaN_3_ was used, the process was inhibited without influencing the adsorption (Fig. SM3). These results suggest that ${{}_{}{}^{1}\mathrm{O}}_{2}$ played the main degrading role in the BRH-FeCl_3_/PMS system.

The literature reports that scavengers such as sodium azide can directly react with PMS, providing false positive results for ROS (Lee et al. 2020). Hence, to confirm the results obtained with the scavenger tests, EPR analyses were also carried out. Figure 4 b–d show that for BRH-FeCl_3_, in the presence of DMPO, DEPMPO, and TEMPO trappings, there were no significant signals for ${\mathrm{SO}}_{4}^{\bullet -}$ and ${\mathrm{HO}}^{\bullet }$, ${\mathrm{O}}_{2}^{\bullet -}$, and ${{}_{}{}^{1}\mathrm{O}}_{2}$, respectively. This indicates that the BRH-FeCl_3_ alone cannot generate ROS (Fig. 4b–d). Therefore, the adducts were evaluated with PMS and BRH-FeCl_3_/PMS. The DMPO-${\mathrm{HO}}^{\bullet }$ and DMPO-${\mathrm{SO}}_{4}^{\bullet -}$ signals in Fig. 4b for PMS and BRH-FeCl_3_/PMS were similar and even overlapped. Consequently, it was confirmed that the system BRH-FeCl_3_/PMS does not produce ${\mathrm{HO}}^{\bullet }$ and ${\mathrm{SO}}_{4}^{\bullet -}$. In turn, Fig. 4c presents signals for the interaction of DEPMPO adduct with PMS alone. However, these signals were inhibited using BRH-FeCl_3_/PMS, which suggests the absence of ${\mathrm{O}}_{2}^{\bullet -}$ in the carbocatalytic process. Meanwhile, the EPR spectrum using TEMP (Fig. 4d), for PMS, showed three characteristic lines of singlet oxygen, which increase their intensity with BRH-FeCl_3_/PMS, confirming the production mainly of ${{}_{}{}^{1}\mathrm{O}}_{2}$ in this carbocatalytic system. In addition to the use of scavengers and EPR analyses, to identify the origin of ${{}_{}{}^{1}\mathrm{O}}_{2}$, an experiment under an N_2_ atmosphere was carried out (Fig. 4a). The results in the N_2_ atmosphere showed no inhibition in ACE degradation, suggesting that ${{}_{}{}^{1}\mathrm{O}}_{2}$ is not generated from the dissolved oxygen in the medium and this ROS effectively comes from the interaction of PMS with the BRH-FeCl_3_.

**Fig. 5:**
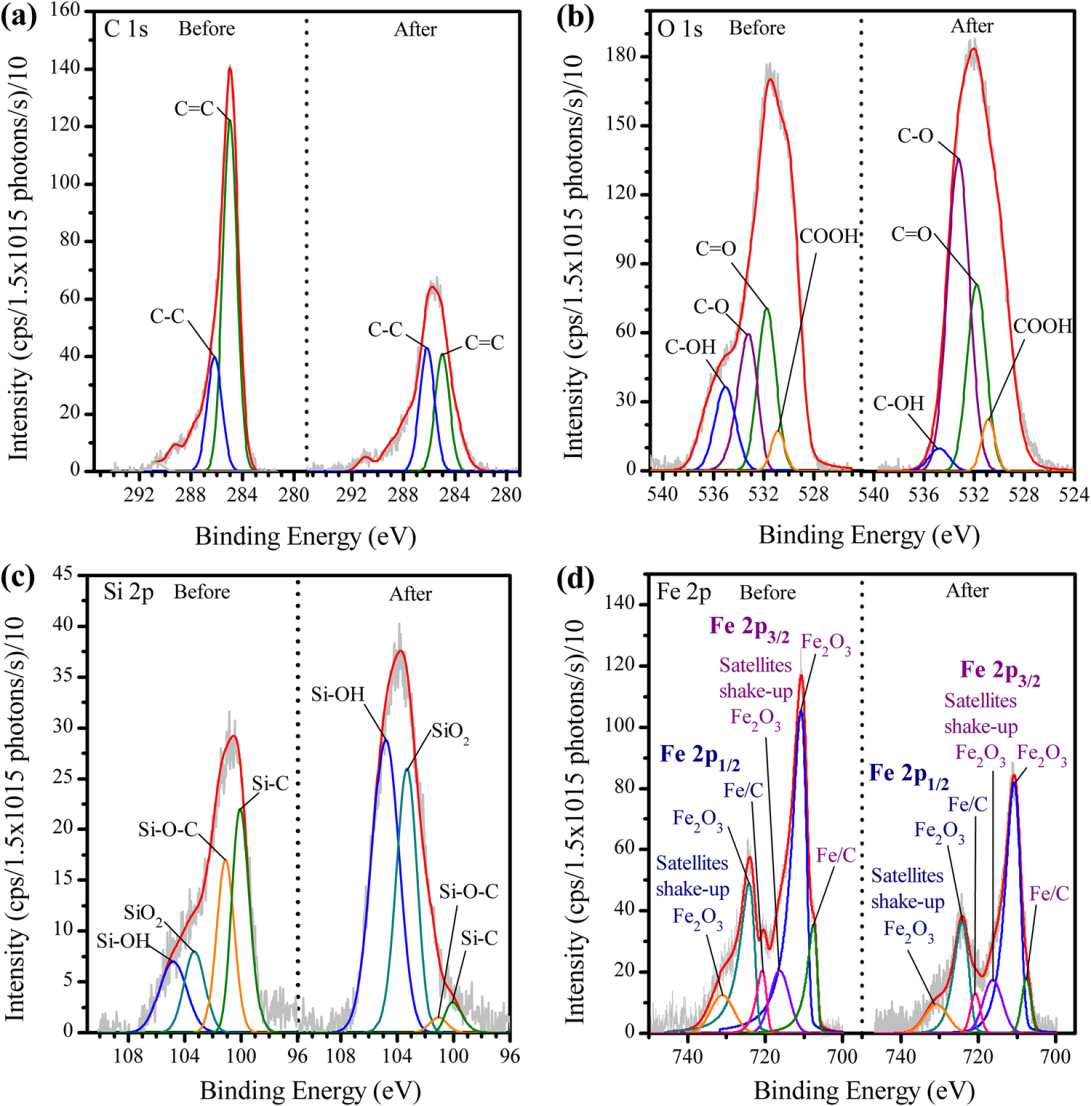
XPS signals deconvolution before and after degradation of ACE **a** C 1s; **b** O 1s; **c** Si 2p; **d** Fe 2p. Conditions: [ACE] = 2.4 mg L^−1^, [BRH-FeCl_3_] = 0.65 g L^−1^, [PMS] = 0.54 mM, pH = 6.8

To unravel the mechanism of the singlet oxygen generation, XPS analyses for BRH-FeCl_3_ before and after ACE degradation, applying the carbocatalysis, were performed (Fig. 5a–d, SM4, and Table SM3). A comparison between the initial XPS spectrum and after degradation evidenced changes lower than 6% in each element for BRH-FeCl_3_, which denotes the high stability of BRH-FeCl_3_. However, these small changes indicate that some oxidation processes are involved in the BRH-FeCl_3_ surface. In fact, before the carbocatalytic process, the deconvolution of C1s for BRH-FeCl_3_ (Fig. 5a) showed 56.6% of C=C (C sp^2^) at a binding energy of 285.00 eV and 19.1% of C–C (C sp^3^) at 286.18 eV (Chen et al. 2020). After the ACE degradation, a decrease in the percentage of C=C (C sp^2^) and an increase in the C–C (C sp^3^) of the BRH-FeCl_3_ was observed. This increase suggests the generation of defects in BRH-FeCl_3_, such as oxygen vacancies (OVs), which can react with the PMS and produce ${{}_{}{}^{1}\mathrm{O}}_{2}$ (Eq. 5) (Zhao et al. 2020; Kohantorabi et al. 2021).

5 $$\mathrm{OVs}+ {2\mathrm{HSO}}_{5}^{-}\to {{}_{}^{1}\mathrm{O}}_{2}+2{\mathrm{HSO}}_{4}^{-}$$

The deconvolution of O 1s in BRH-FeCl_3_ (Fig. 5b) exhibited functional groups such as COOH (530.88 eV), C=O (531.76 eV), C-O (533.22 eV), and C–OH (535.05 eV) (Mandal et al. 2019; Huang et al. 2021b; Xi et al. 2021) with percentages of 2.9%, 15.7%, 15.7%, and 9.3%, respectively. However, after the interaction of BRH-FeCl_3_ with PMS, C–OH decreased, which could be due to oxidations on the surface of the PCM, as reflected by an increase of functional groups such as C=O, C-O, and COOH. It must be considered that deprotonated carboxylic acid species (COO^−^) can interact with PMS, forming the sulfur pentoxide anion (Eq. 6). The sulfur pentoxide anion attacks PMS, and subsequently, ${{}_{}{}^{1}\mathrm{O}}_{2}$ is produced (Eq. 7) (Lee et al. 2020).

On the other hand, according to the ash determination, BRH-FeCl_3_ has a high content of silicon (“Evaluation of the ability of PCMs to activate PMS for the degradation of ACE”). Then, the XPS peak for Si 2p was deconvolved (Fig. 5c). The analysis for BRH-FeCl_3_ before carbocatalysis showed that 30.0% is Si–C (100.07 eV), 23.2% corresponds to Si–O-C (101.11 eV) (Mandal et al. 2019), 12.9% is SiO_2_ (103.30 eV), and 14.9% belongs Si–OH (104.82 eV). However, after the carbocatalytic process, a drastic decrease in Si–C and Si–O-C was obtained because these functional groups were oxidized by the PMS action, producing SiO_2_ and Si–OH groups, respectively. The Si–OH group has a pKa of around 4.5 (Leung et al. 2009), and at the experimental pH (6.8), it is deprotonated. Then, the -Si–O^−^ moiety can also experience an acid/base reaction with the PMS, generating a sulfur pentoxide anion (Eq. 8), and subsequently, ${{}_{}{}^{1}\mathrm{O}}_{2}$ is produced (Eq. 7) (Lee et al. 2020; Serna-Galvis et al. 2023). Thereby, the BRH-FeCl_3_ is an ashes-rich material that has -Si–O^−^ moieties able to generate ${{}_{}{}^{1}\mathrm{O}}_{2}$, and this ROS is very efficient for the degradation of ACE.

6 $$-{\mathrm{COO}}^{-}+ {\mathrm{HSO}}_{5}^{-}\to -\mathrm{COOH}+ {\mathrm{SO}}_{5}^{2-}$$

7 $${{\mathrm{SO}}_{5}^{2-}+\mathrm{ HSO}}_{5}^{-}\to {{}_{}{}^{1}\mathrm{O}}_{2}+{{\mathrm{SO}}_{4}^{2-}+\mathrm{ HSO}}_{4}^{-}$$

8 $$-{\mathrm{Si}-\mathrm{O}}^{-}+ {\mathrm{HSO}}_{5}^{-}\to -\mathrm{Si}-\mathrm{OH}+ {\mathrm{SO}}_{5}^{2-}$$

Regarding the iron species in the non-used BRH-FeCl_3_, we should consider Fe 2p (Fig. 5d), it has two peaks for Fe^3+^ associated with the presence of Fe_2_O_3_. The first peak is Fe 2p_3/2_ at 710.76 eV with a content of 46.1% and the other binding energy is at 724.76 eV with a percentage of 21.4% related to Fe 2p_1/2_ (Xiong et al. 2019). The peaks at 716.26 eV and 731.06 eV are the satellite shake-up of Fe_2_O_3_ in Fe 2p_3/2_ and Fe 2p_1/2_, respectively (Huang et al. 2021b). BRH-FeCl_3_ also shows Fe^0^ represented as the Fe/C functional group, which is observed 10.3% in Fe 2p_3/2_ (707.56 eV) and 5.6% in Fe 2p_1/2_ (720.76 eV) (Oliver-Tolentino et al. 2013).

9 $${2\mathrm{Fe}}^{0}+3{\mathrm{HSO}}_{5}^{-}\to {\mathrm{Fe}}_{2}{\mathrm{O}}_{3}+ {3\mathrm{HSO}}_{4}^{-}$$

After the degradation process, Fe^0^ decreased, due to its oxidation toward Fe^3+^ on the surface by the interaction with PMS, thus increasing Fe_2_O_3_. Iron oxidation could be rationalized considering a direct interaction between Fe/C and PMS with no formation of radicals, leading to the binding of oxygen to iron (Eq. 9). This proposal was confirmed with an additional experiment using H_2_O_2_ (Fig. SM5). Such an experiment showed that the combination of BRH-FeCl_3_ with H_2_O_2_ removed a percentage of ACE similar to that reported by the adsorption process alone. Even the H_2_O_2_ did not react with the iron leached (~ 0.44 mg L^−1^) from the material. In conclusion, Fe in the surface and leached from BRH-FeCl_3_ did not activate H_2_O_2_ (or even PMS) toward the production of radicals (which typically involves electrons transfer from the metal toward the peroxide species; Li et al. 2022). Despite the ferric ions in the lattice of Fe_2_O_3_ could interact with PMS, forming sulfur pentoxide radical anion (which also can evolve into the singlet oxygen), this reaction is slow (Xiao et al. 2020). Hence, all the results suggested that iron had a very relevant role in the synthesis as a structuring substance of the material, but it has low participation in the carbocatalytic process. It is important to mention that our results contrast with those reported in previous work about the degradation of ACE by an iron-doped bone char, in which iron species are more involved in the production of ROS (such as radicals, superoxide radical anion, and singlet oxygen) (Zeng et al. 2023).

### Primary transformations of ACE and toxicity of the treated solution

**Fig. 6:**
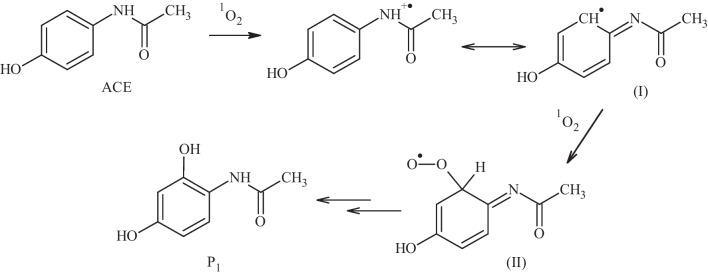
Proposed degradation pathway of ACE using BRH-FeCl_3_/PMS to form P_1_

The primary transformations by the action of the BRH-FeCl_3_/PMS system on ACE were also studied. LC–MS analyses were performed, and one primary degradation product (P_1_) was identified (Figs. SM6 and SM7). Figure 6 shows the chemical structure of P_1_. The same intermediate (P_1_) has also been found as a primary product during the treatment of ACE by electrocatalysis, using a graphitic carbon nitride anode and multiwall carbon nanotube co-doped with Ti/PbO_2_ and photo-electrocatalysis using a praseodymium-polyethylene glycol-PbO_2_//Ti//TiO_2_-nanotubes electrodes, involving the attack of ${\mathrm{HO}}^{\bullet }$ radical species (Chen et al. 2021b; Zhao et al. 2021). Also, an analogous product (i.e., hydroxylation at the aromatic ring on ACE) was informed during the degradation of ACE by an iron-doped bone char (Zeng et al. 2023). It is important to mention that the formation of P_1_ in our system can be explained considering an initial attack of the singlet oxygen to the nitrogen atom on ACE (which is the moiety richest in electron density, as supported by atomic charge analysis in Fig. SM8 (Sehnal 2022)). The initially formed species from ACE experiences a radical delocalization on the aromatic ring (I), and this can react with another molecule of singlet oxygen generating (II), which subsequently evolves to P_1_ (Fig. 6).

**Fig. 7:**
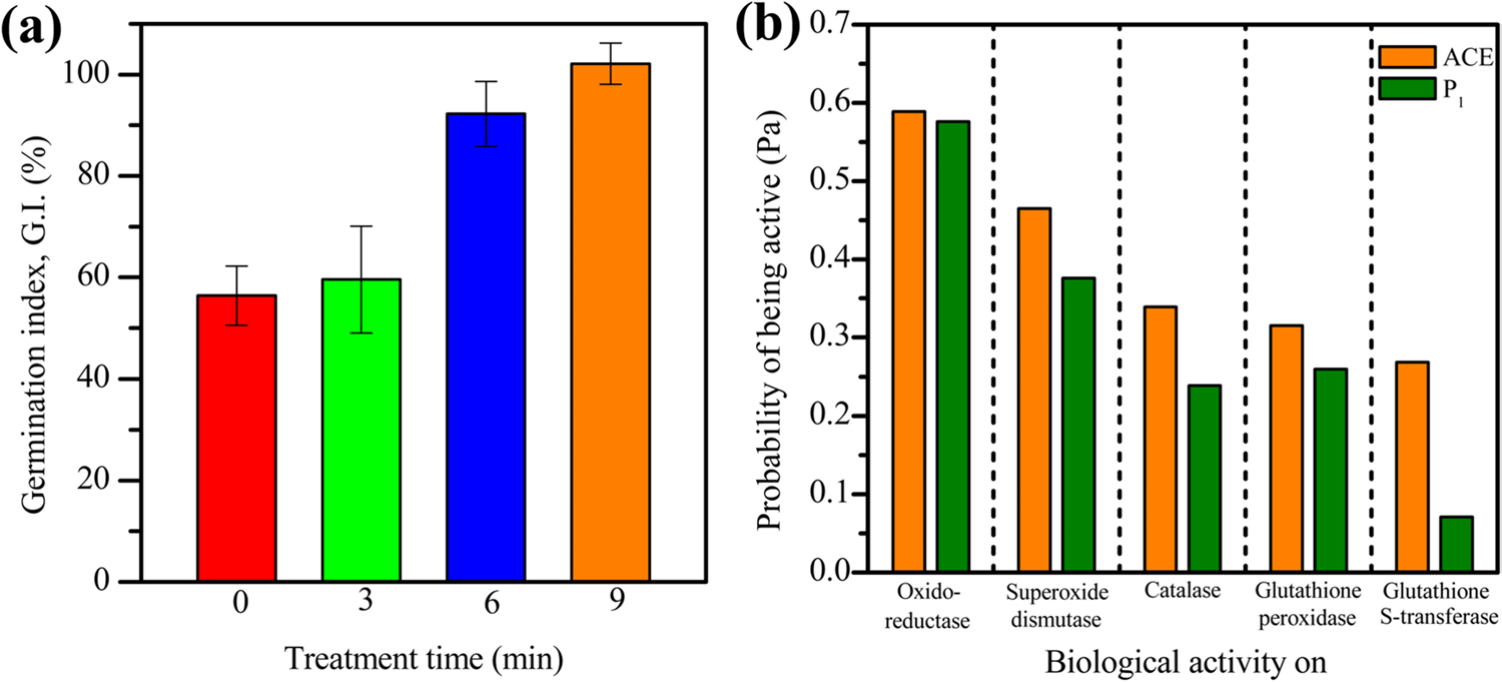
Toxicity analysis of ACE and degradation by-product (P_1_) using BRH-FeCl_3_/PMS. **a** Phytotoxicity of the treated solution against seeds of mung bean and **b** biological activity prediction for ACE and P_1_

Once the structure of P_1_ was elucidated, the phytotoxicity of the resultant solution, which contains this transformation product, was analyzed. The G.I. values obtained for the test using mung bean (Bożym 2022) are presented in Fig. 7a. The results show that after 3 min of treatment, the phytotoxicity is similar to the non-treated sample. However, extending the treatment time up to 6 min or 9 min produces non-phytotoxic solutions. Besides the phytotoxicity tests, computational tools were used to predict other characteristics of P_1_. Thus, the susceptibility to biodegradation of this product was analyzed using the predictive tool from EAWAG (EAWAG 2022). Therefore, the aerobic transformation pathways of ACE and P_1_ were obtained and compared (Fig. SM9). The theoretical results for the aerobic biodegradability showed that the transformation product has a greater number of likely biodegradation pathways than ACE (i.e., P_1_ has a higher probability of being biotransformed than its parent pollutant). In addition to the biotransformation analyses, the biological activity of ACE and P_1_ was studied through the PASS software (W2D Team—PharmaExpert 2021). The probabilities of being active (Pa) for the parent analgesic and its primary transformation product are shown in Fig. 7b. It should be noted that ACE has a high Pa for the biological activity of oxidoreductase, superoxide dismutase, catalase, glutathione peroxidase, and glutathione S-transferase, which could induce negative effects on living organisms (see Table SM4).

Interestingly, P_1_ exhibited Pa values lower than the ACE for each of the considered activities (Fig. 7b), suggesting that the carbocatalytic treatment of ACE produced a substance with lower biological effects than the parent pharmaceutical. Hence, the above results indicate that the carbocatalytic degradation of ACE generated a non-phytotoxic solution and a by-product with lower biological activity (a less toxic compound probably) and more biodegradable than the target pollutant, thus representing a positive environmental impact of the treatment by the BRH-FeCl_3_/PMS system.

### Performance of the BRH-FeCl_3_/PMS system at different pH and in a complex matrix

**Fig. 8:**
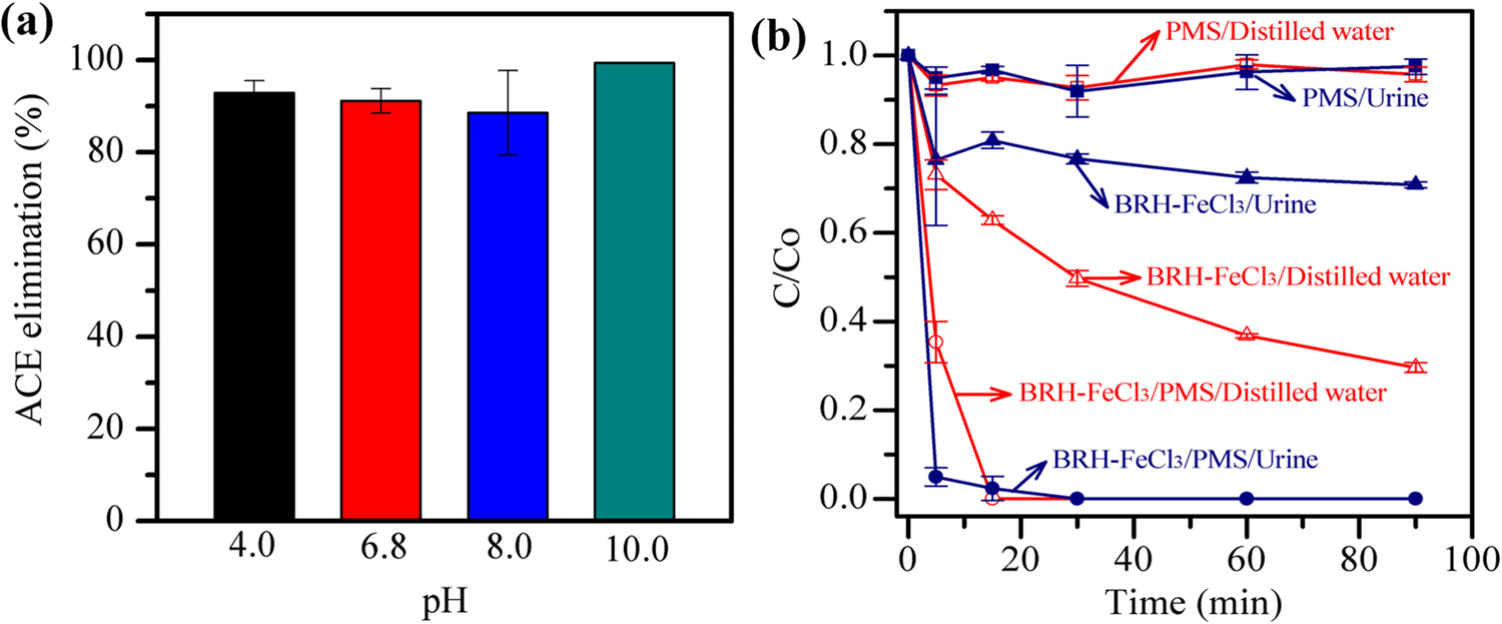
Potential applications of BRH-FeCl_3_ with PMS in the elimination of ACE. **a** pH effect in a range of 4–10. Conditions: [ACE] = 2.4 mg L^−1^, [BRH-FeCl_3_] = 0.65 g L^−1^, [PMS] = 0.54 mM. **b** Matrix effect such as distilled water and urine at pH 5.8. Conditions: [ACE] = 2.4 mg L^−1^, [BRH-FeCl_3_] = 0.2 g L^−1^, [PMS] = 0.5 mM

Another key factor in chemical oxidation using carbocatalysis is the pH (Nguyen et al. 2021). Thus, the efficiency of BRH-FeCl_3_/PMS to degrade ACE under different initial pH conditions was evaluated (Fig. 8a). High ACE eliminations (≥ 88%) were found at the different pH values after only 3 min of treatment. The highest synergy of the process was obtained at pH 6.8, whereas the lowest synergistic effect was observed at pH 10.0 (Fig. SM10). These results can be explained by means of the structural changes of PMS and the surface charge of the BRH-FeCl_3_ under the work conditions.

Under acidic conditions (pH 4), BRH-FeCl_3_ (which has a PZC of 5.8; Table 1) is positively charged. Thus, some species on the material are protonated, limiting their reactions with PMS (Eqs. 6–8), consequently decreasing ROS production and reducing ACE degradation efficiency.

In the case of alkaline conditions (i.e., pH = 8.0 and 10.0), the sulfur pentoxide anion (${\mathrm{SO}}_{5}^{2-})$ is predominant (Xiao et al. 2020) and the BRH-FeCl_3_ material is negatively charged (according to its PZC value). Then, electrostatic repulsive forces are generated, which limit the interaction between the BRH-FeCl_3_ and PMS, and thus, the ACE degradation by the carbocatalytic route is affected at basic pH. At the near-neutral pH (i.e., 6.8), ~ 90% of charges in BRH-FeCl_3_ are negative, while 10% are positive (estimation based on the PZC of this material). Therefore, the interaction between -COO^−^/-Si–O^−^ on BRH-FeCl_3_ and PMS is plausible, producing singlet oxygen (Eqs. 6 and 7), which is the main responsible for the highest degradation of ACE and process synergy observed at pH 6.8.

On the other hand, during the illness treatments, ~ 10% of ACE intake is excreted in the urine without any modification (Aminoshariae and Khan 2015); in the present study, the degradation of ACE in urine was considered. Thereby, simulated urine was used to systematically investigate matrix effects on ACE degradation by using the BRH-FeCl_3_/PMS system. Figure 8 b presents that the sole action of PMS, in both distilled water and simulated urine, only removed 8.0% of ACE at 90 min of exposure to the oxidant. Subsequently, the adsorption of the pollutant was studied, showing that BRH-FeCl_3_ adsorbed 70% of ACE in distilled water, while in simulated urine, the removal decreased to 29% after 90 min. The matrix effect is associated with the urea present in the urine matrix, which competes with ACE for the adsorption sites on the carbonaceous material (Paredes-Laverde et al. 2019).

Interestingly, the action of the BRH-FeCl_3_/PMS system resulted in a very high ACE elimination in the urine matrix (Fig. 8b). A pharmaceutical removal close to 100% in distilled water and urine was achieved within 15 min of treatment. Surprisingly, at shorter treatment times (e.g., 5 min), the ACE degradation was considerably better in urine (79%) than in distilled water (37%). From Fig. 8b, it can also be noted that the adsorption is affected by the urine matrix, while the degradation is considerably increased regarding the distilled water. Indeed, the process synergy in urine was 4, which doubles the synergetic value reported in distilled water (Fig. SM11).

To unravel the role of urine, additional carbocatalytic experiments were performed evaluating the effect of each urine component on the degradation of ACE, at the same concentration of the complex matrix (Fig. SM12). Interestingly, the results showed that in the presence of KCl, ACE can be eliminated in only 3 min; whereas with the other matrix components (CaCl_2_, MgCl_2_, NH_4_Cl, NaH_2_PO_4_, Na_2_SO_4_, or urea), the pollutant elimination required longer treatment times. The superior performance in the presence of KCl can be associated with the transformation of ${\mathrm{Cl}}^{-}$ into HOCl by the oxidation with ${\mathrm{HSO}}_{5}^{-}$ (Eq. 10). The HOCl has strong oxidizing properties, and this agent is able to degrade ACE (Eq. 11; Deborde and von Gunten 2008). Also, the decomposition of HOCl can generate extra ${{}_{}{}^{1}\mathrm{O}}_{2}$ (Eqs. 12–13; Zhang et al. 2020; Lin et al. 2021) useful to enhance the ACE degradation. Furthermore, the carbonaceous material could react with the hypochlorous acid, producing other ROS (Eq. 14) that may increase the elimination of the pollutant (Voudrias et al. 1986).

10 $${\mathrm{HSO}}_{5}^{-}+{\mathrm{Cl}}^{-} \to {\mathrm{SO}}_{4}^{2-}+\mathrm{ HOCl}$$

11 $$\mathrm{HOCl}+\mathrm{ ACE }\to \mathrm{ degradation\;products}$$

12 $$2\mathrm{HOCl }\to {}^{1}{\mathrm{O}}_{2}+2{\mathrm{H}}^{+}+2{\mathrm{Cl}}^{-}$$

13 $${2\mathrm{HSO}}_{5}^{-}+2\mathrm{HOCl }\to {}^{1}{\mathrm{O}}_{2}+ {2\mathrm{Cl}}^{-}+ {2\mathrm{SO}}_{4}^{2-}+ {2\mathrm{H}}_{2}\mathrm{O}$$

14 $${\mathrm{BRH}-\mathrm{FeCl}}_{3}+\mathrm{HOCl }\to \mathrm{ROS}$$

All the results presented above advise that the BRH-FeCl_3_/PMS combination could be a system of interest for the ACE removal to be applied at scales larger than the laboratory context. Nevertheless, before large applications, an important aspect to consider is the electric energy consumption (EEC) of the carbocatalytic process (Zhang et al. 2016). Then, EEC for ACE elimination in distilled water and urine by the BRH-FeCl_3_/PMS process was calculated (details are shown in “Electric energy consumption” and Text SM2). The EEC for the ACE elimination in urine (12.81 kWh m^−3^) presented a value close to the obtained in distilled water (16.67 kWh m^−3^; Table SM5). Finally, it must be mentioned that these EEC values indicated that our carbocatalytic system belongs to AOPs having low-moderate energy consumption (1–100 kWh m^−3^) (Miklos et al. 2018), suggesting a high feasibility of BRH-FeCl_3_/PMS to be implemented for degrading ACE in aqueous samples.

## Conclusions

Two pyrogenic carbonaceous materials (i.e., BRH and BRH-FeCl_3_) were effectively synthesized and evaluated in a carbocatalytic process using PMS for the ACE elimination in water. BRH-FeCl_3_ adsorbed ACE (in 15 min) faster than BRH (which did it in 90 min). Thus, the efficiency of BRH-FeCl_3_ was associated with the higher value of D_AP_ (3.41 nm) and a high content in functional groups such as -COO^−^ or -Si–O^−^ and oxygen vacancies (OVs) according to FTIR and XPS analysis, which allowed to remove 100% of pollutant by adsorption and degradation. Moreover, BRH-FeCl_3_ promoted a higher activation of PMS toward the degrading species process. It is important to remark that iron had a very relevant role in the synthesis as a structuring substance of the PCM, but it has low participation in the carbocatalytic process directly. Interestingly, BRH-FeCl_3_ can be efficiently reused during 4 cycles and was also able to activate PDS leading to ~ 53% of ACE degradation but exhibited better results for degrading the model pharmaceutical in the presence of PMS (synergistic index of 2.4). The experimental design showed that PMS concentration and BRH-FeCl_3_ dose had the predominant influences on pollutant elimination, and an excess of PMS or PCM leads to scavenging reactions that slow down the elimination of ACE. Then, optimization of PMS and BRH-FeCl_3_ amounts maximized the pharmaceutical elimination (~ 93% in only 3 min with low reagents consumption).

The PMS activated with BRH-FeCl_3_ produced ^1^O_2_ (the non-radical route), which was the main responsible for ACE degradation, producing a primary transformation product (P_1_), which presented a hydroxylation on the aromatic ring. Moreover, the resultant solutions are non-phytotoxic (indeed, the test against mung beans showed that after only 6 min of treatment, the phytotoxicity is removed). Also, theoretical analyses of biotransformation and biological activity of P_1_ indicated that this product is more susceptible to aerobic biodegradation and less active biologically (i.e., it could have low toxic effects) than its parent compound. Moreover, the carbocatalytic system was able to partially oxidize (COD removal 37%) and mineralize (DOC removal 19%) the target pollutant. Finally, BRH-FeCl_3_/PMS was an efficient system to degrade ACE in a wide range of pH (4–10) and a complex matrix such as urine. Also, BRH-FeCl_3_/PMS demonstrated a superior efficiency carbocatalytic in urine with an ACE degradation of 70%, which might be related to the presence of the ${\mathrm{Cl}}^{-}$ ions that promoted the formation of HOCl and its interaction with the PCM, leading to extra degrading species. Furthermore, the electric energy consumption of BRH-FeCl_3_/PMS for the degradation of ACE in urine showed a low-moderate value (12.81 kWh m^−3^), suggesting the high feasibility of this treatment system to be implemented for degrading ACE in complex aqueous samples.

### Supplementary Information

Below is the link to the electronic supplementary material.

Supplementary file1 (DOCX 4.87 KB)

## Data Availability

Data and materials will be available upon request to the authors.
