# Supplementary material for: Rice husk–based pyrogenic carbonaceous material efficiently promoted peroxymonosulfate activation toward the non-radical pathway for the degradation of pharmaceuticals in water
Source: Environ Sci Pollut Res Int. 2023 Nov 22;30(59):123616–32. doi: 10.1007/s11356-023-30785-1 (PMC10746782; doi:10.1007/s11356-023-30785-1)
Supplement: Supplementary file 1 — Supplementary file1 (DOCX 4.87 KB) [file 11356_2023_30785_MOESM1_ESM.docx]

**Supplementary Information**

**Rice husk-based pyrogenic carbonaceous material efficiently promoted peroxymonosulfate activation toward the non-radical pathway for the degradation of pharmaceuticals in water**

Marcela Paredes-Laverde^1^, Jazmín Porras^2^, Nancy Acelas^3^, Jhonnaifer J. Romero-Hernández^1^, Sindy D. Jojoa-Sierra^1^, Lázaro Huerta^4^, Efraím A. Serna-Galvis^1,5^, Ricardo A. Torres-Palma^1,^*

*^1^ Grupo de Investigación en Remediación Ambiental y Biocatálisis (GIRAB), Instituto de Química, Facultad de Ciencias Exactas y Naturales, Universidad de Antioquia UdeA, Calle 70 No. 52-21, Medellín, Colombia.*

*^2^ Grupo de Investigaciones Biomédicas Uniremington, Facultad de Ciencias de la Salud, Corporación Universitaria Remington (Uniremington), Calle 51 No. 51-27, Medellín, Colombia.*

*^3^ Grupo de Materiales con Impacto, Mat&mpac, Facultad de Ciencias Básicas, Universidad de Medellín, Medellín, Colombia.*

*^4^ Instituto de Investigaciones en Materiales, Universidad Nacional Autónoma de México, A.P. 70-360, Ciudad de México 04510, México.*

*^5^ Grupo de Catalizadores y Adsorbentes (CATALAD), Instituto de Química, Facultad de Ciencias Exactas y Naturales, Universidad de Antioquia UdeA, Calle 70 # 52-21, Medellín, Colombia.*

******* *Corresponding author: ricardo.torres@udea.edu.co (R. A.T-P.)*

**Text SM 1.** Characterization of BRH-FeCl_3_ by X-ray photoelectron spectroscopy analyses (XPS)

XPS was performed in an ultra-high vacuum (UHV) system Scanning XPS microprobe PHI 5000 VersaProbe II, with an Al K_α_ X-ray source (hν= 1486.6 eV) monochromatic with 100 µm beam diameter, and an MCD analyzer. The XPS spectra were obtained at 45º to the normal surface in the constant pass energy mode (CAE) E_0_ = 117.40 and 11.75 eV survey surface and high-resolution narrow scan. The surface samples were etched for 2 min with 1kV Ar^+^ at 1 µA/mm². Peak positions were referenced to the background silver 3d_5/2_ photopeak at 368.20 eV, having a FWHM of 0.56 eV, and C 1s hydrocarbon groups at 285.00 eV, Au 4f_7/2_ in 84.00 eV central peak core level position corrected by MultiPak PHI software (2015). The elemental composition and deconvolution analyses were done with the PHI Multipak v. 9.6 (2015) and SDP v 4.1 (2004). For elemental composition, the analyzer's transmission function was corrected with the atomic sensitivity factor (ASF) in Multipack and reference materials (oxides, carbides, cellulose, and lignin), results reported by Scofield (Scofield 1976). The ASFs used were Fe2p (2.946), Si 2p (0.368), O 1s (0.733), C 1s (0.314), and N 1s (0.499). The deconvolution analysis in binding energy for the central peak estimated the uncertainty as about 5% (± 0.05 eV) and a chi-square value of less than 1.

**Table SM1.** Design of experiments with the results of ACE elimination (%) obtained experimental and calculated using the equation model proposed in this study. The % ACE elimination was determined at 3 minutes.

| Experiment | PMS Concentration  (mM) | BRH-FeCl_3_ Dose  (g L^-1^) | ACE  Elimination (%)  Experimental | ACE Elimination (%)  Calculated |
| --- | --- | --- | --- | --- |
| 1 | 0.02 | 0.04 | 12.2 | 4.70 |
| 2 | 0.51 | 0.04 | 13.98 | 23.88 |
| 3 | 1 | 0.04 | 25.98 | 23.58 |
| 4 | 0.02 | 0.52 | 66.59 | 72.34 |
| 5 | 0.51 | 0.52 | 95.95 | 91.52 |
| 6 | 1 | 0.52 | 87.56 | 91.52 |
| 7 | 0.02 | 1 | 76.73 | 78.49 |
| 8 | 0.51 | 1 | 98.15 | 97.66 |
| 9 | 1 | 1 | 98.64 | 97.37 |
| 10 | 0.51 | 0.52 | 90.8 | 91.52 |
| 11 | 0.51 | 0.52 | 92.77 | 91.52 |
| 12 | 0.51 | 0.52 | 95.95 | 91.52 |

**Table SM2.** Chemical composition of fresh urine (Serna-Galvis et al. 2021).

| Fresh Urine | |
| --- | --- |
| Concentration [mg L^-1^] | |
| Urea | 16000 |
| NaCH_3_COO | 10250 |
| Na_2_SO_4_ | 2300 |
| NH_4_Cl | 1800 |
| NaH_2_PO_4_ | 2900 |
| KCl | 4200 |
| MgCl_2_*6H_2_O | 370 |
| CaCl_2_*2H_2_O | 510 |
| NaOH | 120 |


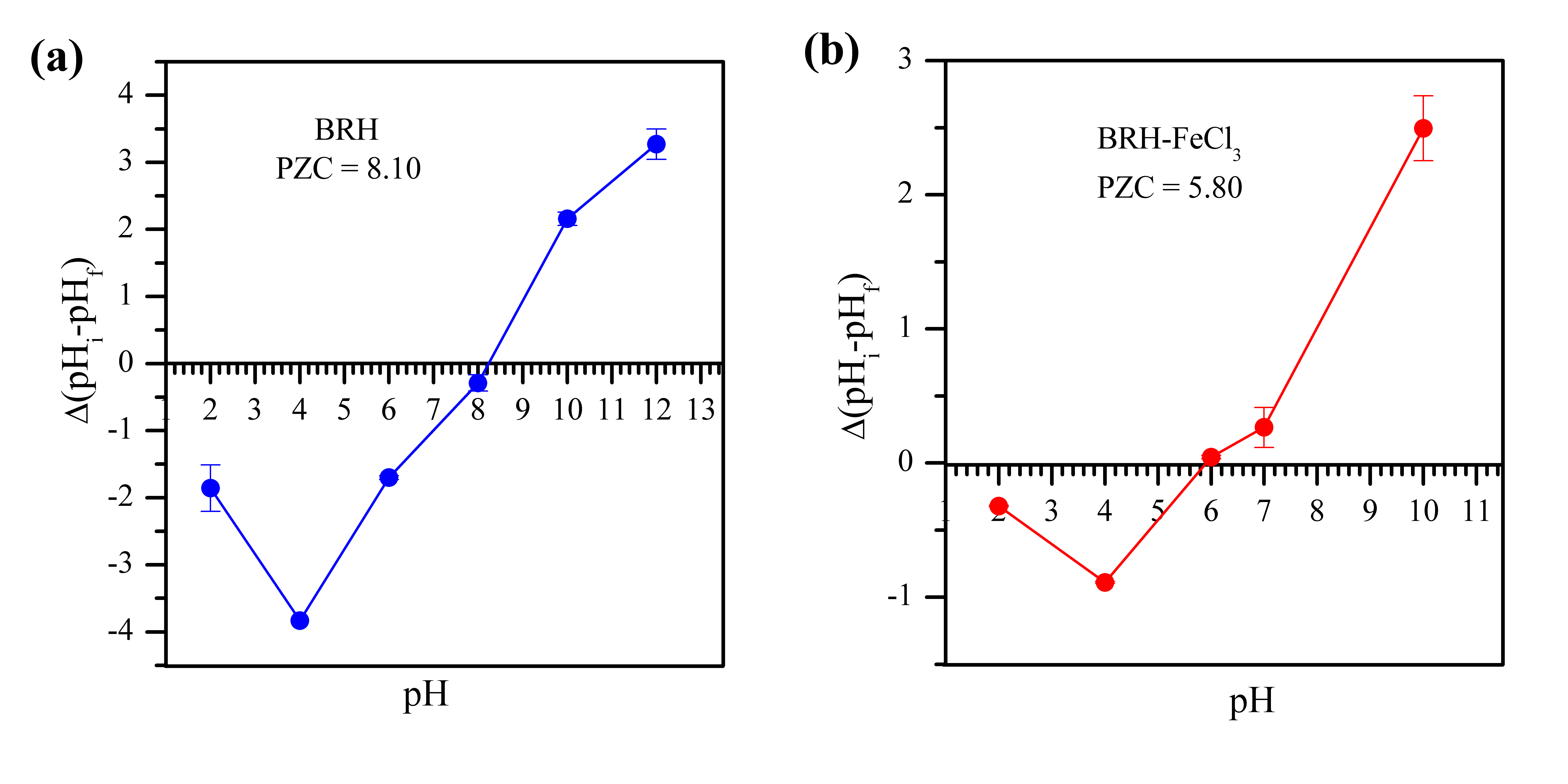


Fig. SM1. Determination of PZC of (a) BRH and (b) BRH-FeCl_3_.





**Fig. SM2.** Synergy of BRH-FeCl_3_ in ACE elimination using PDS and PMS in a time of 15 minutes. Conditions: [ACE] = 2.4 mg L^-1^, [BRH-FeCl_3_] =0.2 g L^-1^, [Oxidizing agent] = 0.5 mM, pH = 6.8.


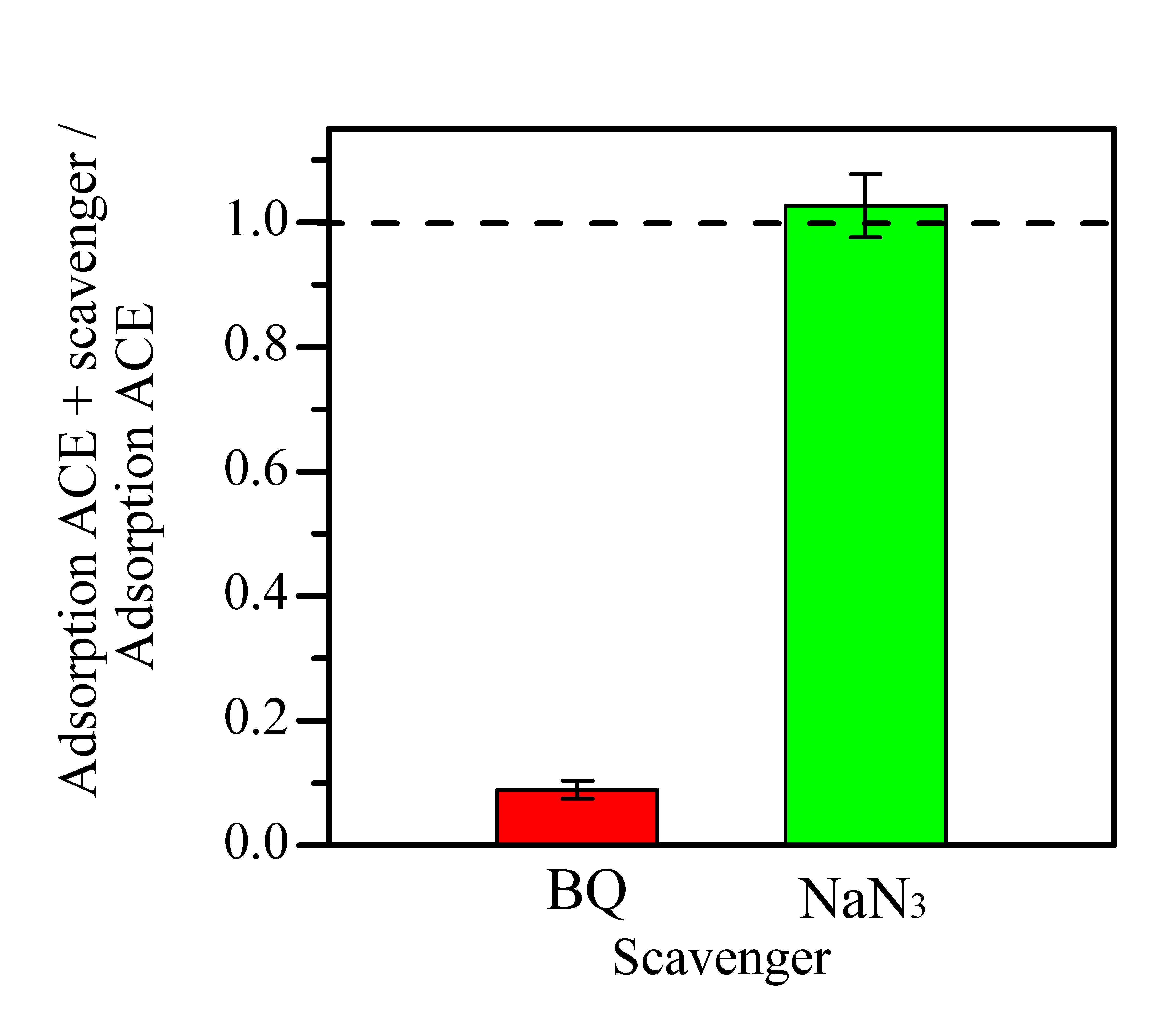


**Fig. SM3.** Adsorption of BQ and NaN_3_ using BRH-FeCl_3_. Conditions: [Scavenger]= 1.6 mM, [ACE]= 2.4 mg L^-1^ (0.016 mM), [PMS] = 0.54 mM and [BRH-FeCl_3_] = 0.65 g L^-1^, time 3 min.





**Fig. SM4.** Survey of BRH-FeCl_3_/PMS before and after use in the ACE elimination.

**Table SM3.** Deconvolution of XPS analysis before and after ACE degradation with BRH-FeCl_3_/PMS.

| Element | Functional group | Fresh BRH-FeCl_3_ | | Used BRH-FeCl_3_ | |
| --- | --- | --- | --- | --- | --- |
|  |  | Binding Energy (eV) | Percentage (%) | Binding Energy (eV) | Percentage (%) |
| C 1s | CH=CH | - | - | 283.70 | 6.8 |
|  | C/Fe | 284.28 | 4.9 | 284.20 | 3.6 |
|  | C-Si | 284.68 | 4.7 | 284.68 | 2.3 |
|  | C=C (C sp^2^) | 285.00 | 56.6 | 285.00 | 25.9 |
|  | C-CH_3_ | - | - | 285.72 | 8.5 |
|  | C-C (C sp^3^) | 286.18 | 19.1 | 286.18 | 28.3 |
|  | C-O | - | - | 287.20 | 8.7 |
|  | C-O-Si | 287.20 | 4.9 | 287.92 | 5.8 |
|  | C-OH | 287.92 | 4.3 | 288.58 | 2.8 |
|  | C=O | 289.26 | 4.2 | 289.26 | 4.1 |
|  | COOH | 290.71 | 1.4 | 290.95 | 3.2 |
| O1s | Fe_2_O_3_ | 530.03 | 40.3 | 530.04 | 30.1 |
|  | COOH | 530.88 | 2.9 | 530.87 | 3.7 |
|  | C=O | 531.76 | 15.7 | 531.80 | 18.6 |
|  | SiO_2_ | 531.78 | 10.5 | 531.77 | 9.3 |
|  | C-O | 533.22 | 15.7 | 533.25 | 35.8 |
|  | C-OH | 535.05 | 9.3 | 534.80 | 2.5 |
|  | Si-OH | 536.48 | 5.6 | 536.52 | 0 |
| Si 2p | Si-C | 100.07 | 30.0 | 100.07 | 3.1 |
|  | Si-O-C | 101.11 | 23.2 | 101.07 | 1.6 |
|  | Si_2_O_3_ | 101.92 | 19.0 | 101.92 | 13.3 |
|  | SiO_2_ | 103.30 | 12.9 | 103.30 | 33.4 |
|  | Si-OH | 104.82 | 14.9 | 104.82 | 48.6 |
| Fe 2p_3/2_ | Fe/C | 707.56 | 10.3 | 707.56 | 6.0 |
|  | Fe_2_O_3_ | 710.76 | 46.1 | 710.76 | 48.6 |
|  | Satellites shake-up Fe_2_O_3_ | 716.26 | 9.6 | 716.26 | 12.1 |
| Fe 2p_1/2_ | Fe/C | 720.76 | 5.6 | 720.76 | 4.6 |
|  | Fe_2_O_3_ | 724.16 | 21.4 | 724.16 | 20.8 |
|  | Satellites shake-up Fe_2_O_3_ | 731.06 | 7.0 | 730.80 | 7.9 |


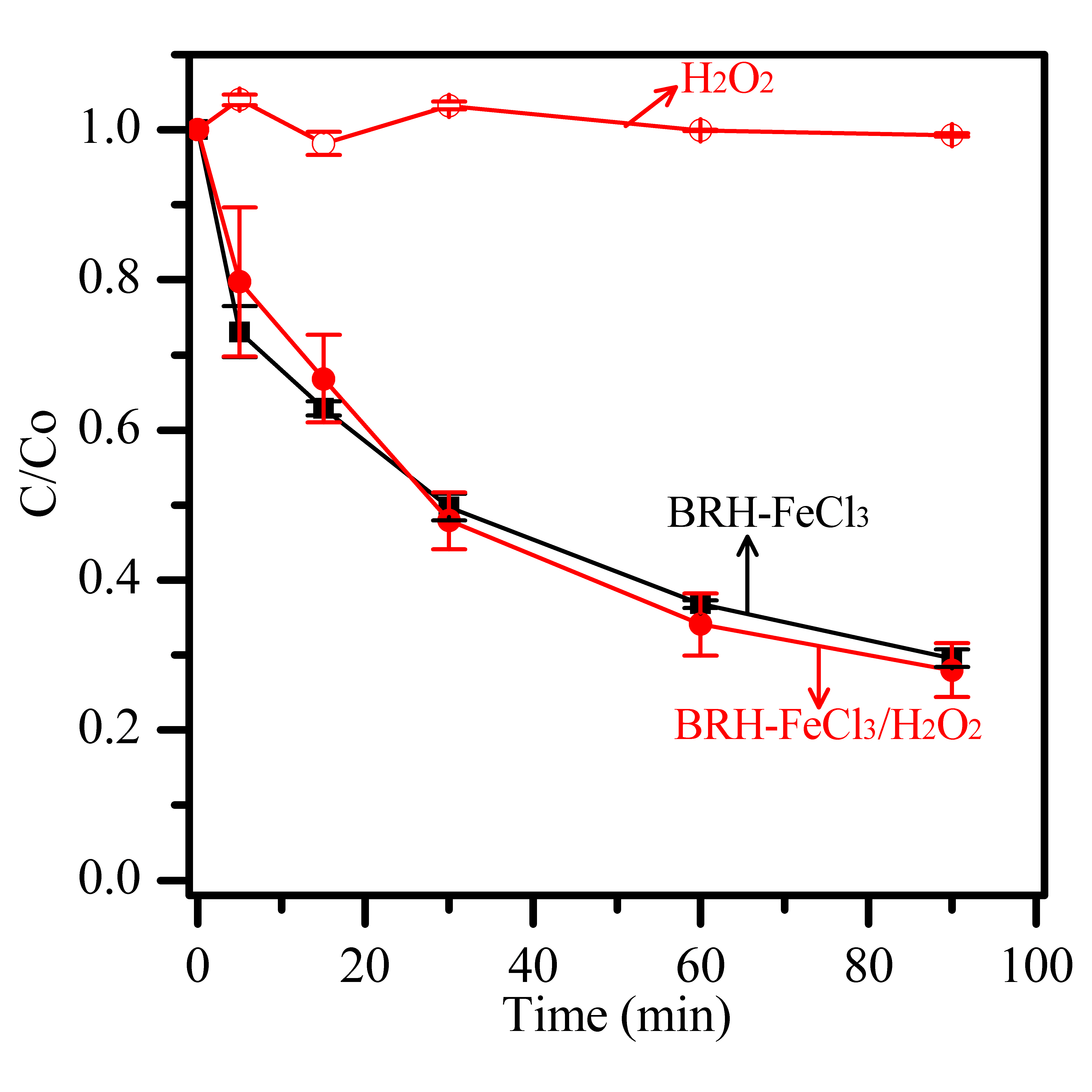


**Fig. SM5.** ACE elimination using BRH-FeCl_3_ with H_2_O_2_. Conditions: [ACE] = 2.4 mg L^-1^, [BRH-FeCl_3_] =0.2 g L^-1^, [Oxidizing agent] = 0.5 mM, pH = 6.8.


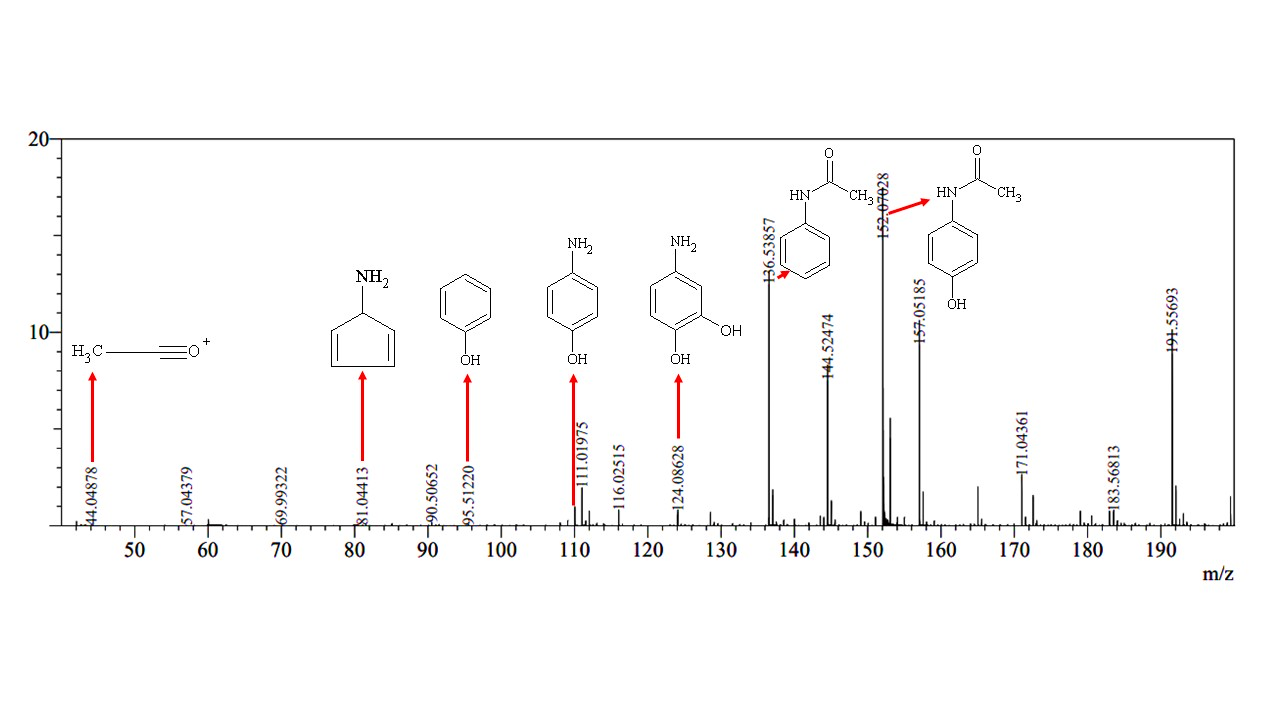


**Fig. SM6.** Mass spectrum of ACE.


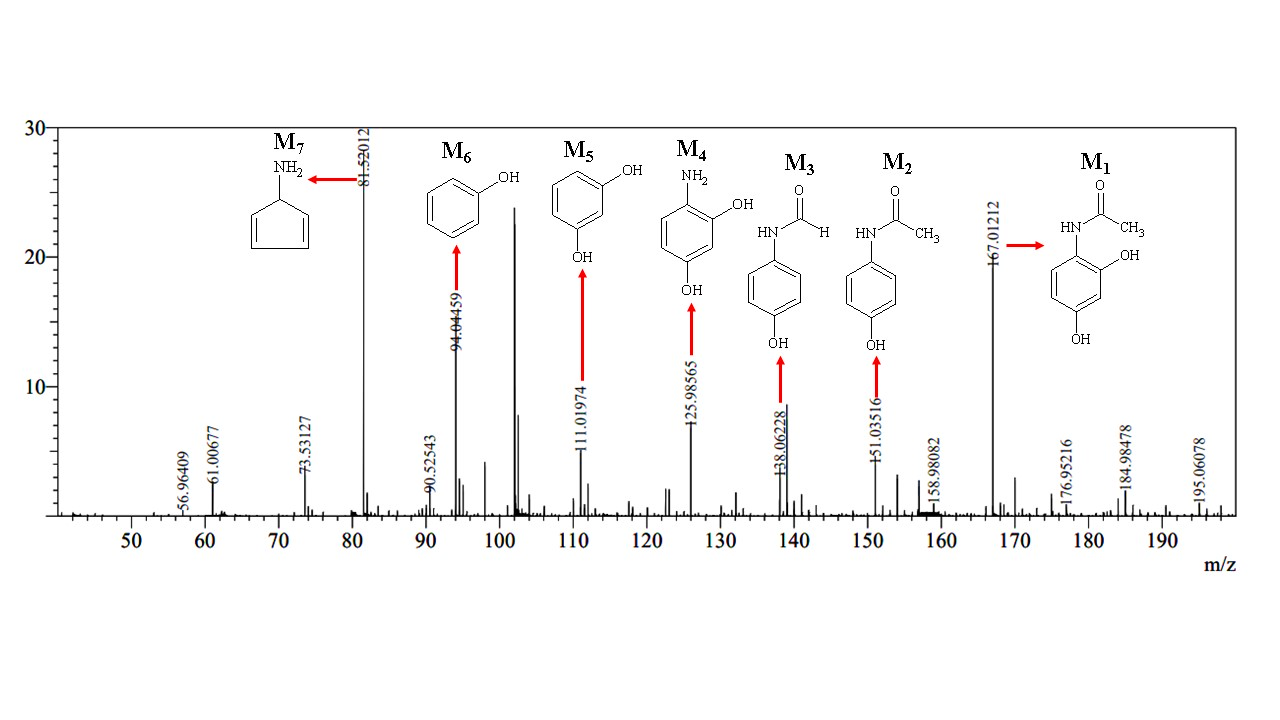


**Fig. SM7.** Mass spectrum of P_1_.


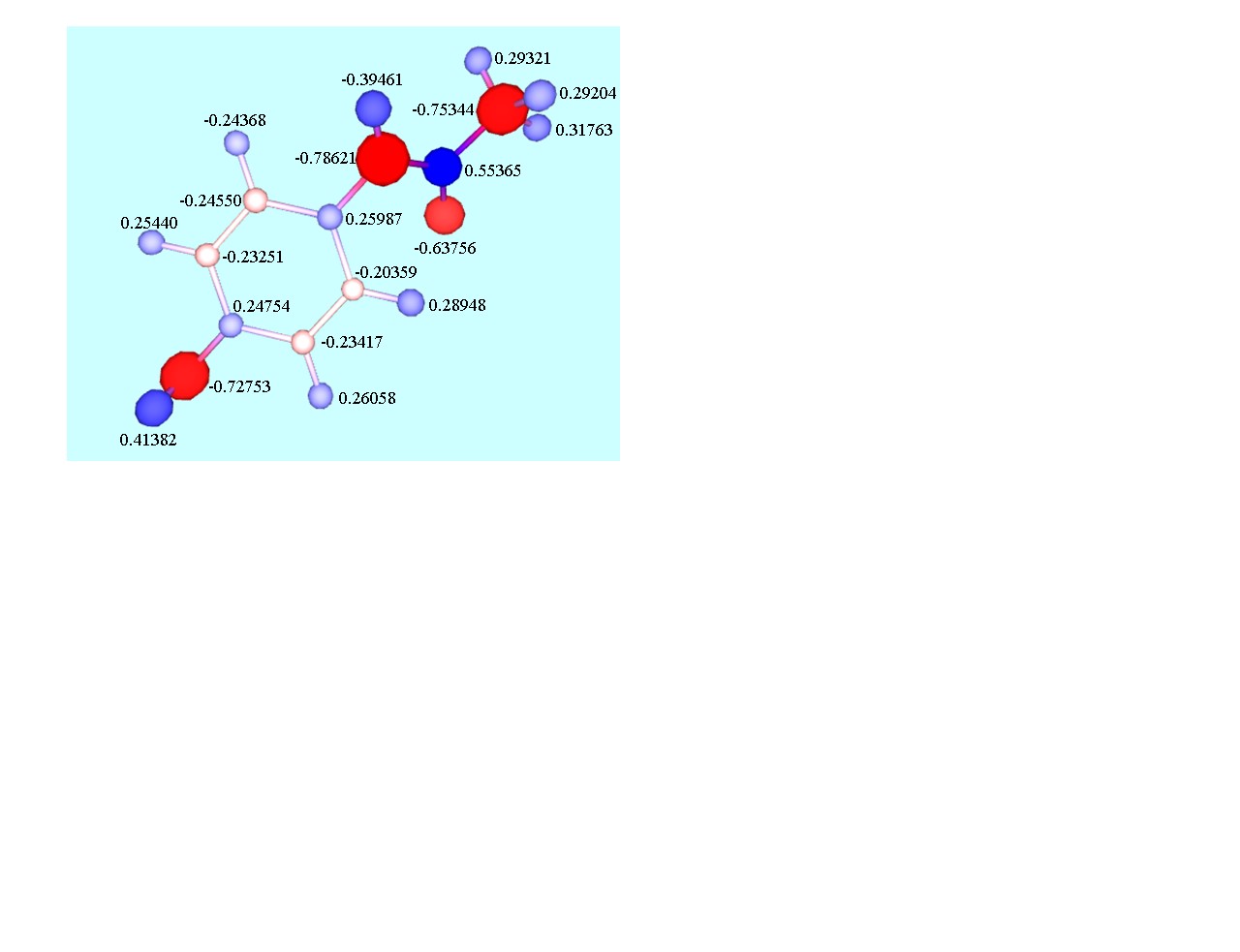


**Fig. SM8.** Atomic charge analysis of ACE using the Atomic Charge Calculator (Sehnal 2022).


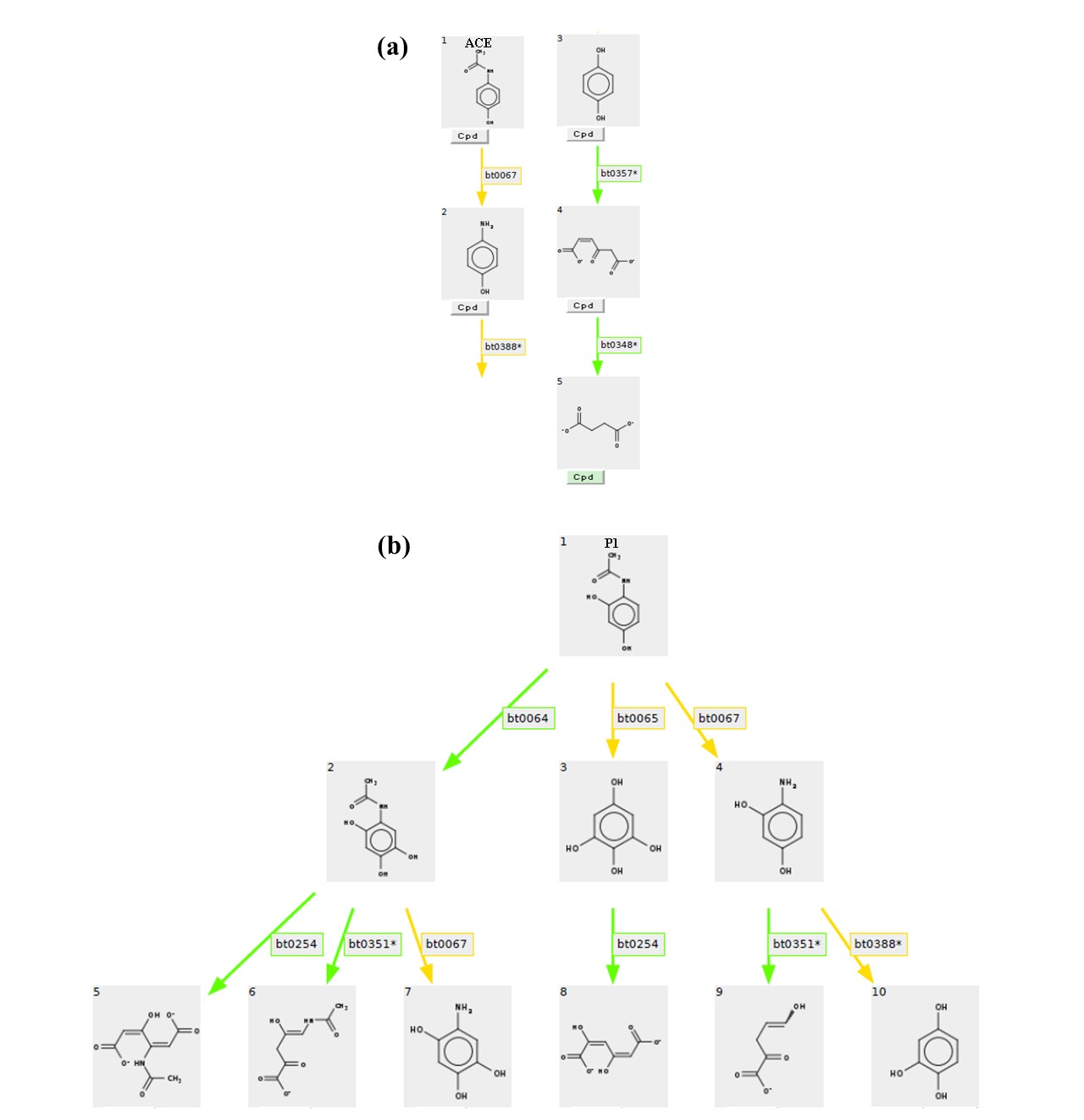


**Fig. SM9.** Prediction of the aerobic transformation pathway of ACE and P1 using Biocatalysis/Biodegradation Database (BBD) from EAWAG (EAWAG 2022). Aerobic Likelihood: Yellow arrows: neutral, green arrows: likely. (a) ACE. bt0067: amide hydrolysis, bt00388*: initial oxidation followed by rapid hydrolysis of the p-aminophenol, bt0357*: cleavage of hydroquinone derivatives, bt0348*: decarboxylation and hydrogenation of double bond. (b) P1. bt0064: hydroxylation of the aromatic ring in the ortho position, bt0065: decarboxylation, hydroxylation of the aromatic ring in the ortho positions, followed by hydrogenation in the meta position of the aromatic ring, bt0067: amide hydrolysis, bt0254: opening the aromatic ring, followed by an E1 elimination in the secondary alcohol, then oxidation of the alkane and the aldehyde group occurs, bt0351*: Opening of the aromatic ring, then an elimination of E1 in the secondary alcohol occurs, followed by oxidation of the alcohol group and finally, a decarboxylation occurs, bt0067: amide hydrolysis, bt0254: aromatic ring cleavage and oxidation, bt0351*; aromatic ring cleavage and oxidation, t0388*: decarboxylation and hydrogenation of double bond.

**Table SM4.** Biological activity of ACE on living organisms.

| **Biological activity on** | **Living organism** | **Reference** |
| --- | --- | --- |
| Oxidoreductase | *Plant Lemna minor* | (Kummerová et al. 2016) |
| Superoxide dismutase | *Hyalella azteca* | (Gómez-Oliván et al. 2012) |
| Catalase | *Hyalella azteca,*  *Marine Polychaete Hediste diversicolor,*  *Brassica juncea L. Czern* | (Bartha et al. 2010; Gómez-Oliván et al. 2012; Daniel et al. 2022) |
| Glutathione peroxidase | *Hyalella azteca* | (Gómez-Oliván et al. 2012) |
| Glutathione S-transferase | *Marine Polychaete Hediste diversicolor,*  *Anguilla anguilla,*  *male fish Rhamdia quelen* | (Nunes et al. 2015; Guiloski et al. 2017; Daniel et al. 2022) |


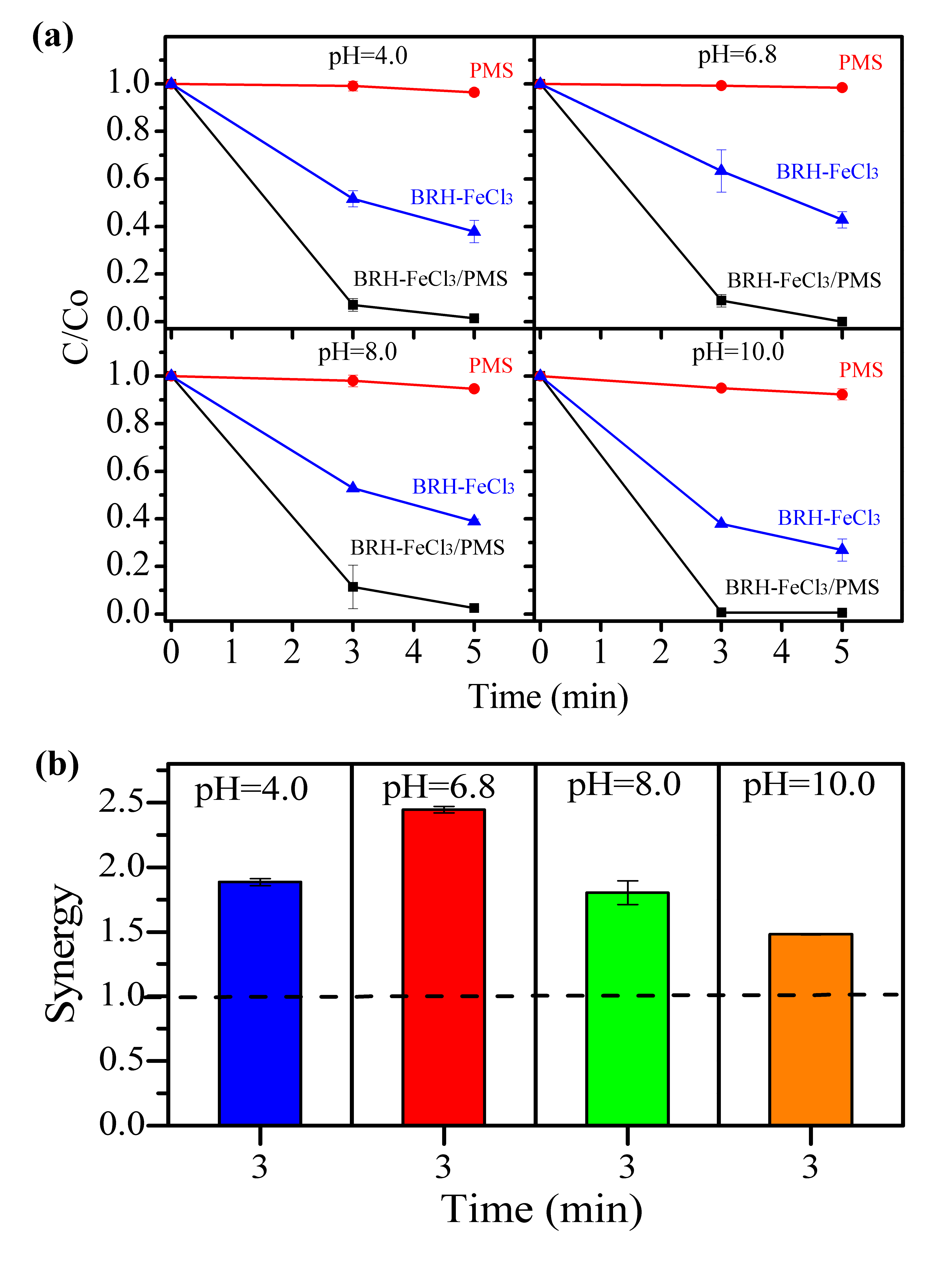


**Fig. SM10.** ACE elimination to different pH of the solution. **(a)** pH effect in a range of 4-10; **(b)** Synergy each pH at 3 min. Conditions: [ACE] = 2.4 mg L^-1^, [BRH-FeCl_3_] =0.65 g L^-1^, [PMS] = 0.54 mM.


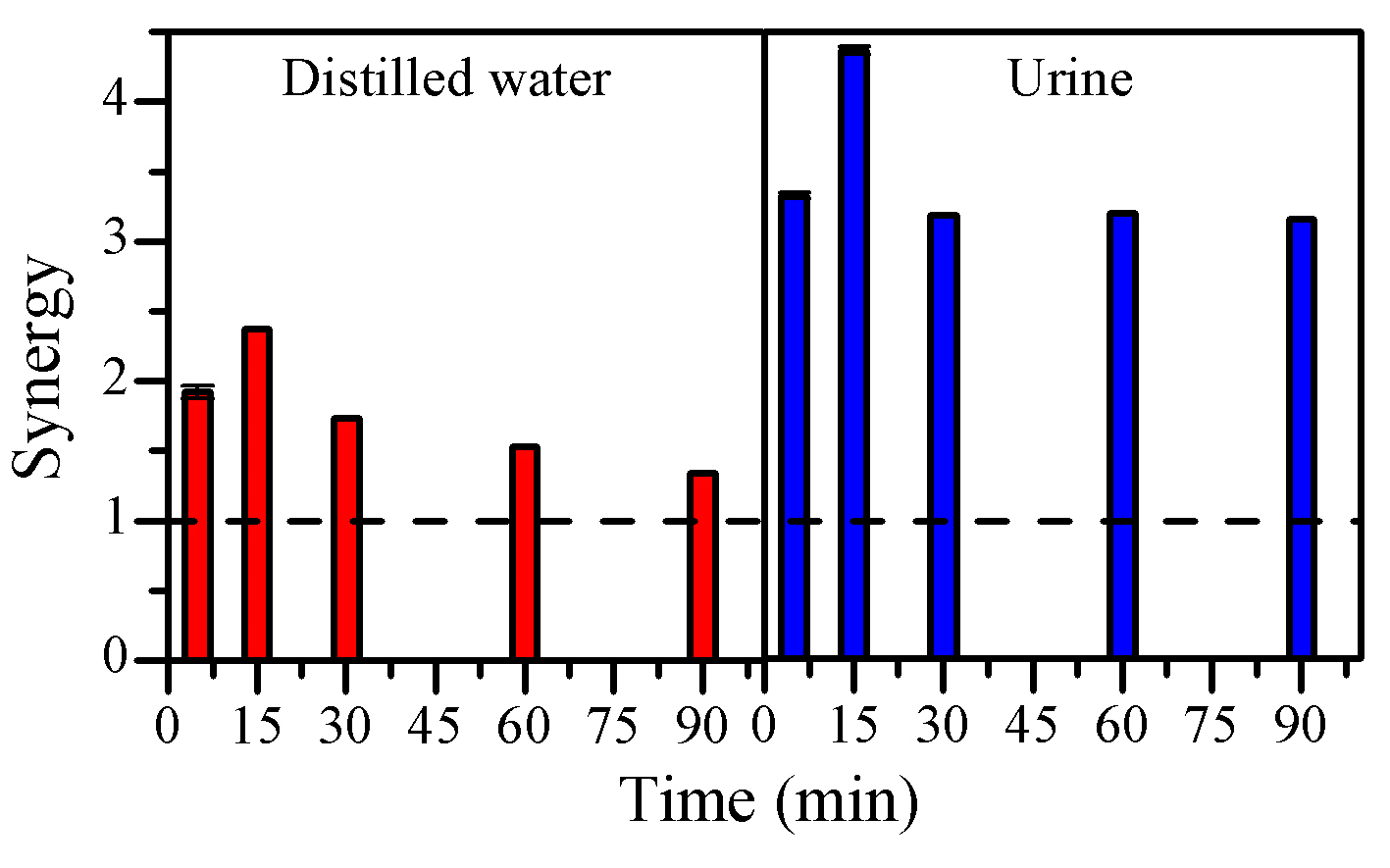


**Fig. SM11.** Synergy in the ACE elimination in a matrix of distilled water and urine. Conditions: [ACE] = 2.4 mg L^-1^, [BRH-FeCl_3_] =0.2 g L^-1^, [PMS] = 0.5 mM, pH = 5.8.


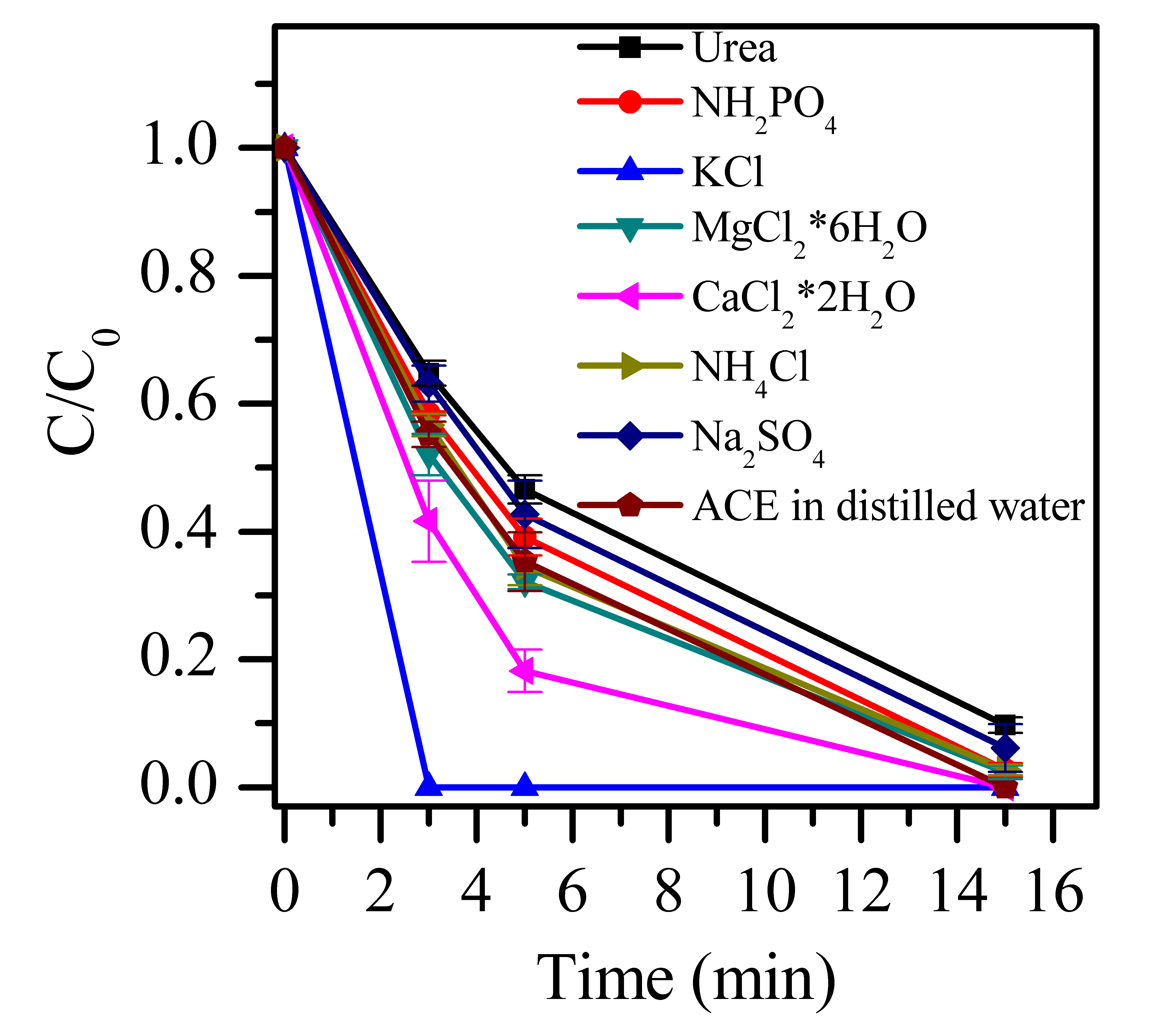


**Fig. SM12.** Effect of the salts of urine in the ACE elimination. Conditions: [Urea] = 16000 mg L^-1^, [NaH_2_PO_4_] = 2900 mg L^-1^, [KCl] = 4200 mg L^-1^, [MgCl_2_*6H_2_O] = 790 mg L^-1^, [CaCl_2_*2H_2_O] = 680 mg L^-1^, [NH_4_Cl] = 1800 mg L^-1^, [Na_2_SO_4_] = 2300 mg L^-1^, [ACE] = 2.4 mg L^-1^, [BRH-FeCl_3_] =0.2 g L^-1^, [PMS] = 0.5 mM, pH = 5.8.

**Text SM 2.** Calculation of electric energy cost (EEC) for BRH-FeCl_3_ in distilled water and urine

- Distilled water

$$EEC= \frac{\left[ P x t x 1000 \right]}{\left[ V x 60 x\log\left( {C_{i}}/{C_{f}} \right) \right]}$$

$$EEC= \frac{\left[ 0.02 kW x 15 min x 1000 L/{m^{3}} \right]}{\left[ 0.1 L x 60 {min}/h x\log\left( \frac{2.4 {mg}/L}{0.0024 {mg}/L} \right) \right]}$$

$$EEC=16.67 kWh m^{-3}$$

- Urine

$$EEC= \frac{\left[ 0.02 kW x 5 min x 1000 L/{m^{3}} \right]}{\left[ 0.1 L x 60 {min}/h x\log\left( \frac{2.4 {mg}/L}{0.12 {mg}/L} \right) \right]}$$

$$EEC=12.81 kWh m^{-3}$$

**Table SM5.** Electric energy consumption (EEC) for BRH-FeCl_3_/PMS.

| **System** | **ACE**  **(mg L^-1^)** | **Power**  **(kW)** | **Time**  **(min)** | **Volume**  **(L)** | **Removal**  **(%)** | **EEC**  $\boldsymbol{(}\boldsymbol{kWh} \boldsymbol{m}^{\boldsymbol{-}\boldsymbol{3}}\boldsymbol{)}$ |
| --- | --- | --- | --- | --- | --- | --- |
| BRH-FeCl_3_ / PMS in distilled water | 2.4 | 0.02 | 15 | 0.1 | 99.9 | 16.67 |
| BRH-FeCl_3_ / PMS in urine | 2.4 | 0.02 | 5 | 0.1 | 95.0 | 12.81 |
| Note: Power was taken from the Dragon Lab magnetic stirrer MS-M-S10 model used in the carbocatalysis experiments. | | | | | | |

**Supplementary references**

Bartha B, Huber C, Harpaintner R, Schröder P (2010) Effects of acetaminophen in Brassica juncea L. Czern.: investigation of uptake, translocation, detoxification , and the induced defense pathways. Env Sci Pollut Res 17:1553–1562. https://doi.org/10.1007/s11356-010-0342-y

Daniel D, Nunes B, Pinto E, et al (2022) Assessment of Paracetamol Toxic Effects under Varying Seawater pH Conditions on the Marine Polychaete Hediste diversicolor Using Biochemical Endpoints. Biology (Basel) 11:1–18. https://doi.org/10.3390/biology11040581

EAWAG (2022) Biocatalysis/Biodegradation database. In: Pathw. Predict. Syst.

Gómez-Oliván LM, Neri-Cruz N, Galar-Martínez M, et al (2012) Assessing the Oxidative Stress Induced by Paracetamol Spiked in Artificial Sediment on Hyalella azteca. Water Air Soil Pollut 223:5097–5104. https://doi.org/10.1007/s11270-012-1261-y

Guiloski IC, Ribas JLC, Piancini LDS, et al (2017) Paracetamol causes endocrine disruption and hepatotoxicity in male fi sh Rhamdia quelen after subchronic exposure. Environ Toxicol Pharmacol 53:111–120. https://doi.org/10.1016/j.etap.2017.05.005

Kummerová M, Zezulkaa S, Babula P, Tríska J (2016) Possible ecological risk of two pharmaceuticals diclofenac and paracetamol demonstrated on a model plant Lemna minor. J Hazard Mater 302:351–361. https://doi.org/10.1016/j.jhazmat.2015.09.057

Nunes B, Verde MF, Soares AMVM (2015) Biochemical effects of the pharmaceutical drug paracetamol on Anguilla anguilla. Env Sci Pollut Res 22:11574–11584. https://doi.org/10.1007/s11356-015-4329-6

Scofield JH (1976) Hartree-Slater Subshell Photoionization cross-sections AT 1254 and 1487 eV. J Electron Spectros Relat Phenomena 8:129–137. https://doi.org/10.1016/0368-2048(76)80015-1

Sehnal D (2022) AtomicChargeCalculator. In: WebChemistry

Serna-Galvis EA, Guateque-Londoño JF, Silva-Agredo J, et al (2021) Ultrasonics Sonochemistry Superior selectivity of high-frequency ultrasound toward chorine containing-pharmaceuticals elimination in urine: A comparative study with other oxidation processes through the elucidation of the degradation pathways. Ultrason Sonochem 80:105814. https://doi.org/10.1016/j.ultsonch.2021.105814

(2015) Multipack version 9.6.0.15, Ulvac-phi, Inc, Physical Electronics USA, 1994-2014

(2004) SDP v4.1 (32 bit) Copyright© 2004, XPS International, LLC, Compiled in January
